# Spatial Distribution of Greenland Shark *Somniosus microcephalus* (Bloch & Schneider, 1801) Life Stages Across the Northern North Atlantic

**DOI:** 10.1002/ece3.71564

**Published:** 2025-06-29

**Authors:** Julius Nielsen, Jørgen Schou Christiansen, Kim Præbel, Peter Rask Møller, Brynn Devine, Klara Jakobsdóttir, Nicolas Straube, Adriana Nogueira, Margaret Treble, Kevin Hedges, Sheila Atchison, Lise Helen Ofstad, Claudia Junge, Laura Wheeland, Rasmus Hedeholm

**Affiliations:** ^1^ Department of Fish and Shellfish Greenland Institute of Natural Resources Nuuk Greenland; ^2^ Natural History Museum of Denmark, University of Copenhagen Copenhagen Denmark; ^3^ Department of Arctic and Marine Biology UiT The Arctic University of Norway Tromsø Norway; ^4^ Norwegian College of Fishery Science UiT The Arctic University of Norway Tromsø Norway; ^5^ Oceans North Halifax Nova Scotia Canada; ^6^ Demersal Division Marine & Freshwater Research Institute Hafnafjörður Iceland; ^7^ University Museum Bergen, University of Bergen Bergen Norway; ^8^ Fisheries and Oceans Canada, Arctic Region Winnipeg Manitoba Canada; ^9^ Faroe Marine Research Institute Tórshavn Faroe Islands; ^10^ Institute of Marine Research Bergen Norway; ^11^ Fisheries and Oceans Canada, Northwest Atlantic Fisheries Centre St. John's Newfoundland Canada

**Keywords:** adult, Arctic, depth, neonate, sleeper shark, temperature

## Abstract

Greenland sharks *Somniosus microcephalus* (Bloch & Schneider, 1801) are long‐lived and highly migratory animals distributed throughout deep and/or cold waters of the North Atlantic Ocean. Extensive bycatch in several demersal fisheries in the Arctic has raised conservation concerns for the species, of which surprisingly little is known about the spatial distribution in relation to their life history. In the current study, size, sex, and life stage composition of 1610 Greenland sharks were examined from 11 geographic regions across the northern North Atlantic Ocean. Subadult females dominated in most regions, and while adult females were scarce or absent in, for example, northern Arctic Canada and Svalbard, they dominated in southwest Greenland and Iceland. Furthermore, in southern Arctic Canada, northwestern Greenland, and southeastern Greenland, adult females were more commonly encountered in offshore waters than inshore. Depth (25 m to 1375 m) had little effect on the spatial distribution irrespective of length and life stage, whereas water temperatures (−1.54°C to 10.9°C) conclusively showed that adult females preferred warm water (> 4°C). Large juveniles were encountered in most regions but dominated in Skagerrak and in offshore southern Arctic Canada. Small juveniles and neonates were encountered with only five and zero records, respectively, combined for all analyzed regions. In an additional effort to identify these rare, small‐sized specimens, scrutinization of museum collections and databases of scientific institutions disclosed a cluster of ten neonates (total length < 60 cm) and two small juveniles (total length 60‐89 cm) along the mid‐Atlantic Ridge and the Irminger Sea. This finding is unique and suggests the location of a potential Greenland shark pupping ground on or in the vicinity of the mid‐Atlantic Ridge and Irminger Sea. All combined, this study provides new insights into the life history of the Greenland shark, which will aid the development of targeted conservation measures.

## Graphical abstract

This study analyzes the body length and sex composition of Greenland sharks across the northern North Atlantic and demonstrates distinct demographic distribution patterns. Areas with high occurrence of adult females, juveniles, or neonates are identified allowing for pinpointing locations of high biological importance for the species listed as “Vulnerable” on the IUCN Red List of Threatened Species.

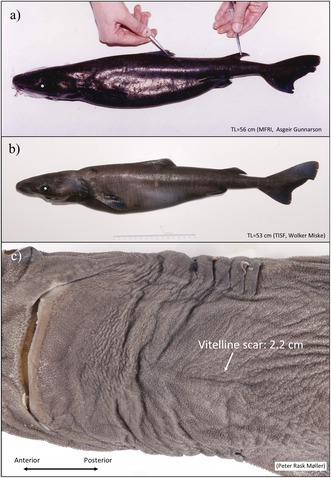

## 1 Introduction

The Greenland shark *Somniosus microcephalus* (Bloch & Schneider, 1801) is among the largest and most widely distributed fish species in the North Atlantic Ocean, including large parts of the Arctic (Mecklenburg et al. 2018; Orlov and Orlova 2024). Throughout the Arctic, they are caught as bycatch in demersal deep‐sea fisheries mainly targeting Greenland halibut *Reinhardtius hippoglossoides* and Atlantic cod *Gadus morhua* (Davis et al. 2013; Edwards et al. 2019; ICES 2021a; Yan et al. 2022). Bycatch issues, combined with an exceptional age at maturity (> 100 years for females, Nielsen et al. 2016) and an expected decline in population size, have recently caused Greenland sharks to be recategorized from “Near Threatened” to “Vulnerable” on the International Union for Conservation of Nature (IUCN) Red List (Kulka et al. 2020).

Despite high bycatch levels and historical high fishery targeting their oily livers (Nielsen 2018), fundamental information about the Greenland shark, and especially life history data, across the full distribution range, is sparse. To improve general management and future conservation efforts, it is important to understand the fundamental biology of the species, which has improved greatly over the past two decades as the Greenland shark has been subject to increased scientific interest. Today, the Greenland shark is well‐established as a highly migratory, slow‐swimming, generalist species mainly feeding on epibenthic fishes and seals (Leclerc et al. 2012; Nielsen et al. 2016, Nielsen et al. 2019a; Watanabe et al. 2012). Although they may tolerate subzero water temperatures, they typically occupy water masses from 0°C to 5°C–6°C, at depths from the surface to at least 2.9 km in the Arctic and North Atlantic (Campana et al. 2015; Fisk et al. 2012; MacNeil et al. 2012; Porteiro et al. 2017). Their capability to perform long‐distance migrations (> 1000 km) has been demonstrated in Canada, Greenland, and Svalbard, and although seasonal residency has also been described, no clear behavioral trends or migration patterns in terms of sex, size, or life stage have been identified (Campana et al. 2015; Edwards et al. 2022; Fisk et al. 2012; Hansen 1963a). In general, observations about their life history are sporadic and scattered in small phrases throughout the scientific literature. Based on observations from West Greenland, it was once proposed that the absence of adult and gravid females could be ascribed to gestation and parturition occurring at great depths in offshore waters beyond fishing activities (Hansen 1963b). More than fifty years later, Campana et al. (2015) supported this view, as the pelagic occurrence of three very large, and likely sexually mature females over abyssal waters off Newfoundland was suggested to be linked to reproduction. The only reported nursery area for juveniles is around Baffin Island (Arctic Canada), where 1–2 m long specimens have been caught over several years (Hussey et al. 2015). To date, only a single gravid Greenland shark with fetuses has been documented throughout the North Atlantic Ocean (Koefoed 1957), and dedicated mating and pupping areas remain unknown.

The purpose of this study is to describe spatial distribution patterns according to body size and associated life stages of male and female Greenland sharks, which will improve the current understanding of’ the species' life history and allow for identification of biological key sites. This will be achieved from length‐based analyses of data acquired from scientific surveys, government fishery observers, scientific literature, and semi‐professional anglers. This approach was possible due to recent advances in the reproductive biology of Greenland sharks, where aspects such as length‐at‐birth, sex‐specific length‐at‐maturity, and maximum length of both males and females have been quantified (see Nielsen et al. 2020).

## 2 Methods

**FIGURE 1:**
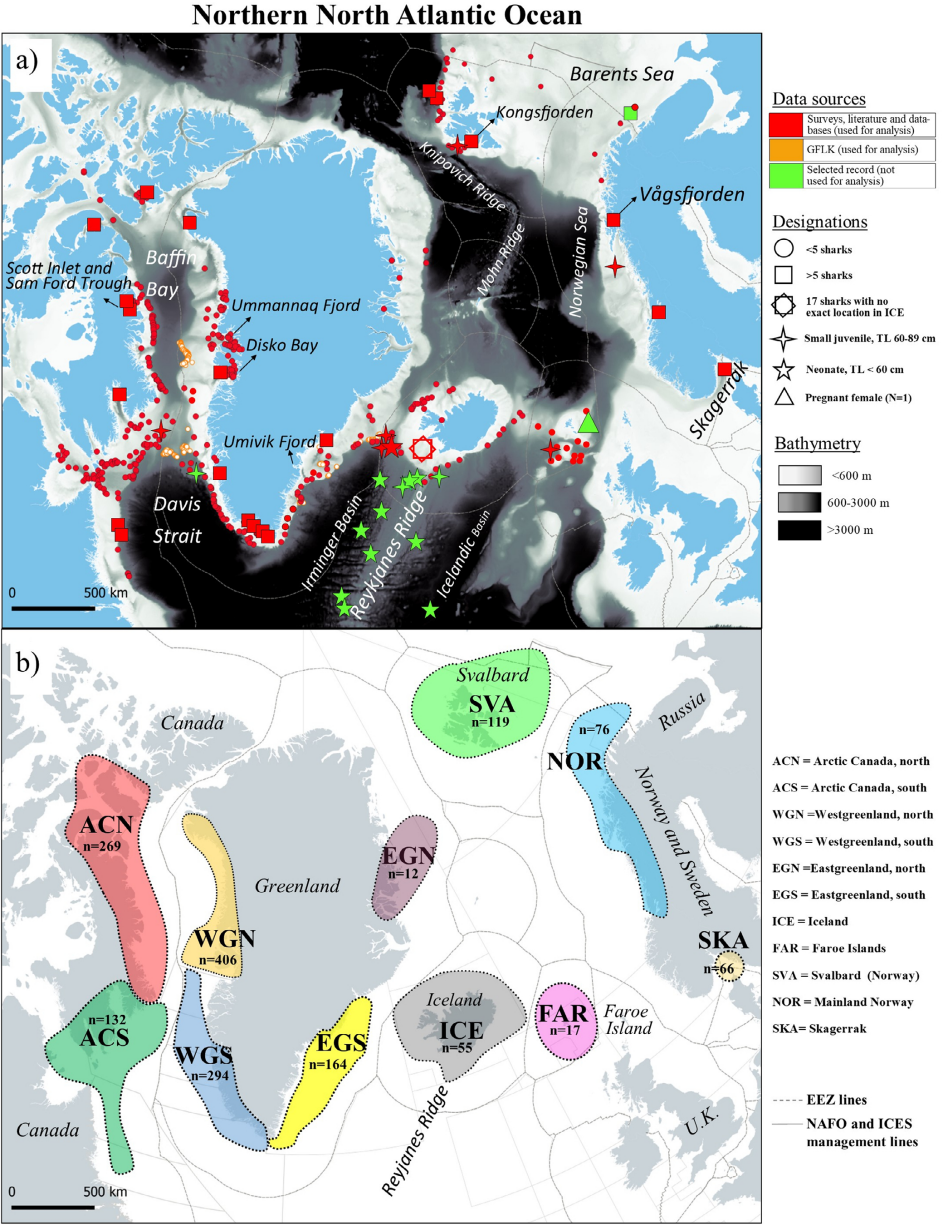
(a) Capture locations of analyzed sharks across the northern North Atlantic. Records from the scientific literature, recreational fishermen and scientific institutions are marked with red, whereas reports from observers of Greenland Fishery and License Office (GFLK) are marked with orange. Selected records (green) represent the Additional Greenland sharks that are not used for statistical analyses but are included for discussion purpose. Total length= TL. (b) The eleven analyzed regions across the northern North Atlantic shown with their respective sample size (*n*). Fishery management boundaries for Northwest Atlantic Fisheries Organization (NAFO) and International Council for the Exploration of the Sea (ICES), as well as the Economic Exclusive Zones (EEZ) are shown.

Data on length, date, gear, and location (exact position or approximate area) were available for 1610 Greenland sharks from Canada, Greenland, Iceland, the Faroe Islands, Norway, and Sweden sampled primarily in the summer and fall, over the past three decades (Figure 1a). For a subset of these, data on sex, depth, and water temperature were available (Tables 1 and S1). The data set was acquired from a range of sources, which can be divided into four overall categories: “Survey databases,” “Other scientific databases,” “Scientific literature,” and “Anglers” (Table 1).

**TABLE 1:** Overview of data sources and sample size for total length (TL), sex, depth (m), and temperature (°C).

Data source (sampling years)	Sample size, *n*				Gear	Region
	TL	Sex	Depth	Temp.		
(1) *Survey databases*						
GINR (1998–2023)	297	194	225	165	T + G + L	WGN, WGS, EGS
DFO (2005–2022)	149	82	118	143	T	ACN, AGS
MFRI (1998–2020)	47	23	47	46	T	ICE, EGS
IMR (1998–2022)	37	9	37	14	T	NOR, SVA
FAMRI (1994–2022)	17	2	17	9	T	FAR
(2) *Other databases*						
GINR shark survey (1996)	93	7	0	0	SL	WGS
GFLK (2019–2022)	362	0	361	0	T	WGN, WGS, EGS
(3) *Scientific literature (see Appendix* S2 *)*						
Campana et al. (2015) (2007–2009)	11	11	0	0	L	ACS
Devine et al. (2018) (2015–2016)	93	93	93	93	C	ACN
Hussey and Devine unpublished data (2020–2021)	37	37	37	0	T	ACN, ACS
Edwards et al. (2022) (2013–2016)^a^	46	46	0	0	SL	ACN
Fisk et al. (2012) (2008–2009)	20	20	0	0	SL	SVA
Hussey et al. (2015) (2011–2013)	54	49	0	0	SL	ACN
Hussey et al. (2018) (2015)	5	5	0	0	SL	ACN
Leclerc et al. (2012) (2008–2009)	45	45	0	0	SL	SVA
McMeans et al. (2010) (2001–2005)^b^	17	17	0	0	T + SL	ICE
Skomal and Benz (2004) (1999)	6	6	0	0	SL	ACN
Nielsen et al. (2019b, 2020) (2012–2018)^c^	154	154	89	0	T + SL	WGN, WGS, EGS, EGN SVA, NOR
(4) *Anglers*						
Thule Shark Busters (2008–2010)	16	0	16	0	F	WGN
Frederic Kullin (2010–2017)	22	21	0	0	F	NOR
Andørja Adventures (2012–2014)	16	3	2	0	F	NOR
Swedish angler records (1999–2022)	66	0	62	0	F	SKA
Total	1610	824	1135	470		

*Note:* Sampling years in parentheses. For all sharks, capture position was available except the 17 sharks of McMeans et al. (2010), which only can be assigned the region Iceland (ICE).

Abbreviations: Gear abbreviations: C, Camera; F, fishing rod with single hook; G, gillnet, L, longline with multiple hooks intended for commercial species; P, pelagic trawl; SL, shark line with multiple hooks intended for Greenland sharks; T, bottom trawl. Institution abbreviations: DFO, Fisheries and Ocean Canada; FAMRI, Faroe Marine Research Institute; GFLK, Greenland's Fisheries License Control Authority; GINR, Greenland Institute of Natural Resources; IMR, Norway Institute of Marine Research; MFRI=Iceland Marine & Freshwater Research Institute. Region abbreviations: ACN, Arctic Canada north; ACS, Arctic Canada south; EGN, East Greenland north; EGS, East Greenland south; FAR, Faroe Islands; ICE, Iceland; NOR, Norway mainland; SKA, Skagerrak; SVA, Svalbard; WGN, West Greenland north; WGS, West Greenland south.

^a^ In the original study *N* = 65, but here *N* = 46 are listed because 19 overlapping specimens were allready reported in Hussey et al. (2015).

^b^ No individual location—only geographical region (ICE).

^c^ Individual sharks are represented multiple times in Nielsen's studies, which have been considered to avoid double reports in the current data set.

### 2.1 Data Sources

“Survey databases” (1994–2023) contains data from 547 specimens obtained from scientific biodiversity and stock monitoring bottom trawl surveys targeting shrimp and groundfish. These surveys have been conducted by the Greenland Institute of Natural Resources (GINR), Fisheries and Oceans Canada (DFO), Marine & Freshwater Research Institute (MFRI) in Iceland, Institute of Marine Research (IMR) in Norway, and Faroe Marine Research Institute (FAMRI). Data from GINR's annual longline and gillnet surveys intended for Greenland halibut and Atlantic cod in Greenland inshore waters were also included among the GINR data (Table 1). The bottom trawl surveys cover depths from 50 to 1500 m in continental shelf and slope waters, and inshore gillnet and longline surveys (in Greenland) cover depths from 165 to 700 m. It should be noted that trawling effort and time span vary between surveys. Specific survey designs and protocols are described for Canada (Doubleday 1981; Fulton et al. 2024; Hedges 2023; Rideout et al. 2022), Greenland (ICES 2023a; Jørgensen 2011; Nogueira et al. 2023; Nogueira and Estévez‐Barcia 2023; Nygaard 2020; Nygaard and Nogueira 2023), Iceland (Jakobsdóttir et al. 2023; Jónsdóttir et al. 2024), Norway, and the Faroe Islands (ICES 2021b) (Table 1).

“Other databases” contain unpublished data for 455 specimens from sampling efforts that targeted Greenland sharks. Data sources include (1) a longline shark test fishery conducted by GINR in 1996 in inshore waters of Southwest Greenland, and (2) data collected by fishery observers from the Greenland Fisheries License Control Authority (GFLK) from 2019 to 2022, where government observers and trained crew recorded lengths onboard three commercial trawlers in Greenland (Table 1).

“Scientific literature” (2004–2022) contains data for 488 specimens from peer‐reviewed studies on Greenland sharks from Canada, Greenland, Iceland, and Svalbard sampled by bottom trawl, specially designed shark longlines, gillnets, and underwater baited cameras (Table 1 and Appendix S2). The included studies were carefully checked, comparing lengths, sampling methods, years, and areas, in order to avoid including duplicate records.

“Anglers” (1999–2022) contains data for 120 specimens acquired by semiprofessional anglers or fishing guides targeting Greenland sharks in Greenland, Norway, and Sweden using baited single hooks. Information on length, capture location, and occasionally depth and sex are available. These data were provided by Thor‐Eivind Flakstad (Andørja Adventures, Norway), Jimmy Bedested (Thule Shark Busters, Greenland), Frederik Kulin (Norway), and Nicka Hellenberg (Swedish register of big game fish “Svensk Storfiskeregister”) (Table 1).

Common for all included data sources is that within the catchable size range of the respective gear types, it is assumed that no further size or sex selection of specimens has been conducted. Consequently, studies focusing on certain shark sizes or sex have not been included as a data source to avoid any potential bias. Notice that 28 additional sharks are included solely for discussion purposes as they violate assumptions for being sampled/recorded randomly in their respective databases (see separate section on additional sharks below). Assumptions of random size/sex sampling, the potential for sampling bias/data skewness, and in particular differences in size‐related selectivity between the different fishing gears that have been used for catching the 1610 sharks are addressed at the end of this Method section and in Appendix S1.

### 2.2 Length Standardization

In this study, all lengths are reported as total length (TL, cm), which is the natural length from snout to upper part of the tail fin in a horizontal straight line. Most of the included data sources report length as TL, although fork length (FL, cm) was reported for 17 specimens from the DFO database and another 17 from McMeans et al. (2010) (see Table 1). To include these 34 sharks in our analysis, their lengths were converted to TL using a species‐specific TL–FL relationship (TL = 10,593 × FL + 4.9387, Nielsen et al. 2014) derived from 47 Greenland sharks. In addition, 56 specimens from the DFO database had only body mass (BM in kg) available. For these, TL was estimated according to a species‐specific length–weight relationship (BM = 4.416 × 10^−6^ × TL^3.1346^, Nielsen et al. 2014). The proportion of samples affected by this TL conversion is low (2% for the FL–TL conversion and 3.5% for the length–weight conversion).

In general, all length measurements of Greenland sharks are associated with some uncertainty because it is notoriously difficult to accurately measure large fish. This is the case for anglers, observers, technicians, and scientists, making this minor bias equal across all data sources. Therefore, we consider the impact of measurement error and the impact of length/weight conversions on the results to be negligible. Any bias is also further reduced as we analyze possible differences between length groups (i.e., life stages), rather than specific lengths (see below).

### 2.3 Geographical Regions and Life Stages

Each of the 1610 Greenland sharks was assigned to one of eleven geographical regions (Figure 1b and Table 2). The delineation of regions is restricted to the continental shelf and slope based on the distribution of capture positions and defined management areas of the Northwest Atlantic Fisheries Organization (NAFO) and the International Council for the Exploration of the Sea (ICES), ICES ecoregions (ICES 2023b), and Exclusive Economic Zones (EEZ):

- ACN = “Arctic Canada north” in NAFO Division 0A, *n* = 269.
- ACS = “Arctic Canada south” in NAFO Division 0B, 2G and 2H, *n* = 132.
- WGN = “West Greenland north” in NAFO Division 1A, *n* = 406.
- WGS = “West Greenland south” in NAFO Division 1B, 1C, 1D, 1E and 1F, *n* = 294.
- EGN = “East Greenland north” in ICES area 14a, *n* = 12.
- EGS = “East Greenland south” in ICES area 14b2, *n* = 164.
- ICE = “Iceland” in ICES area 5a1 and 5a2, *n* = 55.
- SVA = “Svalbard” in ICES area 2b2, *n* = 119.
- NOR = “Norway mainland” in ICES area 2a2, *n* = 76.
- SKA = “Skagerrak” in ICES area 3a20, *n* = 66.
- FAR = “Faroe Islands” in ICES area 5b, *n* = 17.

**TABLE 2:** Data on total length (TL) and capture area (inshore/offshore) are available for all 1610 analyzed specimens, whereas data on sex, depth, and water temperature are only available for a subset.

	TL		Sexed	Depth	Temp	Total
	*n*_Inshore_	*n*_Offshore_	*n*	*n*	*n*	*n*
ACN*	204	65	260	158	131	269
ACS*	32	100	69	121	105	132
WGN*	208	198	156	323	58	406
WGS*	139	155	103	183	75	294
EGN	6	6	11	4	0	12
EGS*	22	142	39	162	41	164
ICE	0	55	38	38	37	55
SVA*	65	54	92	54	0	119
NOR	69	7	54	13	0	76
SKA	0	66	0	62	0	66
FAR	0	17	0	17	9	17
Total	745	865	824	1135	470	1610

*Note:* The number of sharks, *n*, for each parameter is presented below. “Data rich regions” are marked with *.

Abbreviations: ACN, Arctic Canada north; ACS, Arctic Canada south; C, Camera; EGN, East Greenland north; EGS, East Greenland south; FAR, Faroe Islands; ICE, Iceland; NOR, mainland Norway; SKA, Skagerrak; SVA, Svalbard; WGN, West Greenland north; WGS, West Greenland south. Gear abbreviations: F, fishing rod with single hook; G, gillnet; L, longline with multiple hooks intended for commercial fish species; LS, long lines with multiple hooks intended for Greenland shark; T, bottom trawl.

Regions with > 100 sharks are referred to as “Data rich regions” and are evaluated further in terms of length and sex composition in “Inshore” versus “Offshore” waters (Table 3). “Inshore” is defined as waters within 1 NM to the nearest coastline or within a fjord, bay, or inlet.

**TABLE 3:** Sex composition in inshore and offshore waters for the six data‐rich regions (*n*_total_ > 100, cf. Table 2).

	*n*_sexed_	%*F*	Inshore		Offshore	
			*n*_female_	*n*_male_	*n*_female_	*n*_male_
ACN	260	52	98	101	37	24
ACS	69	78	3	14	49	3
WGN	156	64	93	53	7	3
WGS	103	85	39	14	49	1
EGS	39	84	18	4	15	2
SVA	92	58	37	28	16	11
Total	719		288	214	173	44

*Note:* %*F* refers to the proportion of females.

Abbreviations: ACN, Arctic Canada north; ACS, Arctic Canada south; EGS, East Greenland south; SVA, Svalbard; WGN, West Greenland north; WGS, West Greenland south.

To compare regional body size composition, each shark was assigned to one of three length groups:

- Small: TL < 200 cm, *n* = 176
- Medium: TL 200–400 cm, *n* = 1141
- Large: TL > 400 cm, *n* = 293

All sexed sharks (*n* = 824) and unsexed sharks of TL < 200 cm (*n* = 119) were assigned to one of the seven life stages below. The remaining unsexed sharks (*n* = 667) were not assigned to a life stage.

- Neonate: TL < 60 cm (males and females combined), *n* = 1.
- Small juvenile: TL 60–89 cm (males and females combined), *n* = 6.
- Large juvenile: TL 90–200 cm (males and females combined), *n* = 182.
- Subadult male: TL 201–283 cm, *n* = 114.
- Adult male: TL > 283 cm, *n* = 149.
- Subadult female: TL 201–418 cm, *n* = 386.
- Adult female: TL > 418 cm, *n* = 105.

Length ranges for these Greenland shark life‐history categories are based on (1) previously applied body lengths of juvenile and subadults (Edwards et al. 2022; Hussey et al. 2015; Nielsen et al. 2019b); (2) sex‐specific lengths at 50% maturity (TL_50_), that is., TL 284 cm for males and TL 419 cm for females (Nielsen et al. 2020); (3) estimated length‐at‐birth TL ~40 cm (Nielsen et al. 2020; Yano et al. 2007). Note that internal inspection was not performed on the vast majority of the sharks in this study; therefore, we are not able to confirm if any of the large females were gravid.

### 2.4 Data Presentation and Analyses

The length composition for each region was displayed using histograms with 50 cm bin widths, summarized across sampling gears of the respective regions. Proportions of the three length groups and seven life stages are presented for all geographic regions. A regional overview of applied sampling gears and the corresponding numbers of sharks and their size range is presented in Table 4.

**TABLE 4:** Overview of sharks, *n*, and their size range in cm, TL (in parentheses) caught in one of the six different gear types (trawl, gill net, long line, shark line, camera, or fishing rod) in each of the 11 regions.

	*N* (TL range)										
	ACN	ACS	WGN	WGS	EGN	EGS	ICE	SVA	NOR	SKA	FAR
Trawl *N* = 804	67 (124–422)	121 (81–474)	210 (116–490)	155 (106–510)	NA	142 (104–530)	55 (57–480)	30 (85–430)	7 (61–350)	NA	17 (84–500)
Gill net *N* = 78	NA	NA	78 (135–427)	NA	NA	NA	NA	NA	NA	NA	NA
Long line *N* = 13	NA	BA	13 (101–352)	NA	NA	NA	NA	NA	NA	NA	NA
Shark line *n* = 497	109 (100–381)	11 (262–349)	89 (120–450)	139 (170–500)	12 (220–330)	22 (270–386)	NA	89 (245–404)	26 (148–437)	NA	NA
Camera *n* = 93	93 (131–325)	NA	NA	NA	NA	NA	NA	NA	NA	NA	NA
Fishing rod *n* = 125	NA	NA	16 (230–343)	NA	NA	NA	NA	NA	43 (180–440)	66 (109–405)	NA
Total	269	132	406	294	12	164	55	119	76	66	17

*Note:* The expected size selection for each of these gear types is as follows: Trawl = open bottom trawls sample all fish > 5 cm in both benthic and pelagic habitats, gill net = gill net with varying mesh size intended for, for example, commercial Greenland halibut will catch demersal fish from > 40 cm, long line = long lines with small hooks intended for, for example, commercial Greenland halibut will catch demersal fish from > 40 cm, shark line = specially designed long line targeting Greenland sharks with big hooks typically catching sharks from > 150 cm (see Appendix S1), camera = baited underwater video recordings recording all sizes of fauna attracted to the bait, fishing rod = recreational anglers fishing for deep‐sea fishes with single hook sampling fish from > 1 m (see Appendix S1). Abreviations: ACN, Arctic Canada north; ACS, Arctic Canada south; EGN, East Greenland north; EGS, East Greenland south; FAR, Faroe Islands; ICE, Iceland; NOR, mainland Norway; SKA, Skagerrak; SVA, Svalbard; WGN, West Greenland north; WGS, West Greenland south.

Length differences between geographic regions (“Region”) and within regions (i.e., between inshore and offshore areas, “Area”) were compared using a nonparametric Anderson‐Darling k‐sample test and a Kruskal‐Wallis test, respectively (data were not normally distributed). The Anderson‐Darling test is appropriate to apply when more than two groups are compared, while the Kruskal–Wallis test is used for comparing two groups (inshore and offshore). The tests included “Region” and “Area” as factorial variables. To test whether some life stages were more prevalent in certain regions than in others, we compared the distribution of life stages using a chi‐squared test. To explore length at different depths and water temperatures, linear regression models were fitted using “Region” as a factorial variable and “Temperature” or “Depth” as covariates. Two‐way ANOVA and Tukey Post hoc tests were used to explore life stages at different depths and temperatures. To test for sampling gear effects, a three‐way ANOVA corrected for heteroscedasticity by White adjustment (White 1980) was done using “Region,” “Area,” and “Gear” as factorial variables.

All maps were made in QGIS, figures in Sigma plot, and statistical analyses were conducted using R 4.2.3 (R Core Team 2023).

### 2.5 Additional Greenland Sharks

An additional 28 sharks were included for discussion purposes only (Table 5). These sharks were not sampled and/or reported randomly, as they were included in their respective databases due to their small size (see assumptions for included data sources above). Therefore, these records were not used for the statistical analysis, yet they are included in the study as they represent the smallest life stages including the rarely encountered neonates (*n* = 10) and small juveniles (*n* = 8). These two life stages are only represented with one and six specimens, respectively, among the 1610 sharks (see above). Of the 28 additional sharks, four were sampled by commercial pelagic trawlers, six from the German/Icelandic/Russian pelagic surveys targeting beaked redfish *Sebastes mentella* along the Reykjanes Ridge and the Irminger Sea (unpublished data from MFRI and Thünen Institute of Sea Fisheries, TISF, ICES 2015; Dolgov 2015, Dolgov pers. com.), and two were from the scientific litterature (Kukuev and Trunov 2002). There were also 15 specimens from the Barents Sea (unpublished data, University Museum of Bergen) and a single specimen from southwest Greenland (unpublished data Natural History Museum of Denmark) (Table 5). The additional sharks are included strictly for discussion purposes and not included in statistical analyses.

**TABLE 5:** Overview of additional sharks comprised by unpublished records of specimens classed as “Neonate,” “Small juvenile,” or “Large juvenile” based on total length (TL).

Year	TL (cm)	Sex	Depth, capture (m)	Depth, bottom (m)	Position	Source/gear
*Neonates (TL < 60 cm)*					(N; W)	
1981	41.8	NA	NA	2930^a^	55.00; 28.00	Kukuev and Trunov (2002)/PT
1995	55	NA	400	1400^a^	62.83; 26.67	MFRI (survey)/PT
1998	56	M	525	1700	60.03; 26.36	MFRI (survey)/PT
1999	46.7	F	460–800	2550^a^	60.30; 33.70	Kukuev and Trunov (2002)/PT
2001	45.4	NA	560	1570	55.78; 36.15	MFRI (survey)/PT
2001	50	NA	730	2550^a^	63.10; 30.67	MFRI (fisher)/PT
2001	53	NA	770	1800^a^	61.25; 31.13	MFRI (fisher)/PT
2006	51	F	700	1300^a^	62.50; 27.05	MFRI (fisher)/PT
2007	52	NA	600	2200^a^	56.28; 36.38	Pers. com. A Dolgov/PT
2015	53^b^	M	< 1000	2050^a^	58.89; 32.98	TISF (survey/collection)/PT
*Small juveniles (TL 60–89 cm)*						
1992	65^c^	M	1090	1090	63.17; 53.51	NHMDK (survey/collection)/BT
1993	89	NA	700	1165	62.79; 23.19	MFRI (survey)/PT
2006	66	M	720	1300^a^	62.83; 27.00	MFRI (fisher)/PT
					N; E	
2022	80	F	232	232	71.02; 33.15	UiB (collection)/LL
2022	80	F	198	198	71.10; 34.76	UiB (collection)/LL
2022	81	F	199	199	70.88; 33.63	UiB (collection)/LL
2022	82	M	285	285	71.53; 33.73	UiB (collection)/LL
2022	82	M	205	205	71.06; 33.98	UiB (collection)/LL
*Large juveniles (TL 90–200 cm)*						
2022	91	F	207	207	70.93; 34.02	UiB (collection)/LL
2022	92	F	236	236	71.12; 33.80	UiB (collection)/LL
2022	94	F	251	251	71.27; 33.43	UiB (collection)/LL
2022	94	F	241	241	70.93; 32.58	UiB (collection)/LL
2022	100	M	205	205	71.06; 33.98	UiB (collection)/LL
2022	102	F	216	216	70.95; 33.35	UiB (collection)/LL
2022	102	F	225	225	71.05; 33.60	UiB (collection)/LL
2022	102	M	227	227	71.06; 33.72	UiB (collection)/LL
2022	108	M	243	243	71.57; 33.98	UiB (collection)/LL
2022	115	M	208	208	71.03; 34.28	UiB (collection)/LL

*Note:* All records are sampled from pelagic surveys or commercial fisheries using either pelagic trawl (PT), long lines (LL) or bottom trawl (BT). Sex is either female (F) or male (M). Information not available is labeled “NA.” “Fisher” refers to observations made from commercial fisheries, “survey” from scientific surveys and “collection” refers to specimens in museum collections.

Abbreviations: MFRI, Iceland Marine Freshwater Research Institute; NHMDK, Natural History Museum of Denmark; TISF, Thünen Institute of Sea Fisheries (Germany); UiB, University Museum Bergen (Norway).

^a^ Bottom depths acquired from electronic sea charts onboard RV Tarajoq.

^b^ Collection id number: DMM IE/10000.

^c^ Collection id number: ZMUC P07101.

### 2.6 Sampling Gear Differences in Relation to Size‐Selectivity

It is important to address the issue of gear selectivity in terms of size across regions, given different data sources of the 1610 sharks that are statistically analyzed (see also Appendix S1). For example, bottom trawl will catch the full size range of Greenland sharks, whereas long lines designed for catching Greenland sharks (i.e., shark lines) often have large hooks (size 4 or 5), which cannot be assumed to catch the smallest specimens. The majority of data, however, were sampled with gear that has no size selection encompassing bottom trawl (49.9% of sharks), camera (5.8% of sharks), gill nets (4.8% of sharks), and long lines (0.8% of sharks) intended for commercial species (Table 4). Bottom trawls are open nets and will sample both crustaceans and fish from a few centimeters in length up to the maximum length of Greenland sharks and will do so both while towing along the bottom as well as in the pelagic upon gear recovery/deployment. For camera deployments, these are baited and will attract all sizes of sharks. Long lines and gill nets targeting Atlantic cod or Greenland halibut catch fishes from 20 cm to 30 cm in size and can fairly be assumed to catch both small and large Greenland sharks if present—the latter which often gets entangled in such gears (GINR unpublished data, Edwards et al. 2021). Datasets based on sharks caught by these specific gear types can thus fairly be assumed to represent the present sizes of the sampled habitat, albeit not necessarily proportionally to their true abundance, given that some gear types may have a higher selectivity for certain sizes.

The most valuable data source for the purpose of our study is the extensive trawl survey databases, which are available from nine of eleven regions encompassing ACN, ACS, WGN, WGS, EGS, ICE, SVA, FAR, and NOR (Table 4). In these regions, it is of no overall importance for the size comparisons if data are extended also to include catches from, for example, shark lines, which typically target Greenland sharks from 1.5 m and larger (Table 4 and Appendix S1). However, data from EGN and SKA are only from shark lines and fishing rods, respectively. The size composition for these regions could therefore be skewed as small sharks would not be caught by these gears. For EGN, the sample size of only 12 sharks makes any conclusions for this specific region tentative regardless of sampling gear. For SKA, the available sizes of 66 sharks match well the reported sizes over historical time in this particular area of high human activity supporting that the acquired data depicts the true size distribution of the region (see Results and Discussion).

To sum it up, regardless of the difference in sampling gear, the dataset of 1610 sharks (plus 28 additional sharks for discussion purpose) is applicable for the first‐ever comparison between size, sex, and life stages across the Greenland shark's main distribution area.

## 3 Results

### 3.1 Data Overview

Average total lengths of the 1610 specimens were 272 ± 54 cm for males (range 70–375 cm, *n* = 293), 331 ± 84 cm for females (range 57–480 cm, *n* = 531) and 323 ± 96 cm for unsexed specimens (range 61–530 cm, *n* = 786). Of these, 1135 records had information on capture depth, and 470 had information on water temperature (Table 2). Data on length + sex + depth were available for 465 specimens and length + sex + depth + temperature for 294 specimens. For the 11 geographic regions, the number of sharks varied from 12 in EGN to 406 in WGN (Table 2).

### 3.2 Lengths and Life Stages

Sampling gear was unrelated to length (ANOVA, *F* = 1.07, *n* = 1610, *p* = 0.37). Therefore, gear was not considered in subsequent analyses of differences between and within regions.

**FIGURE 2:**
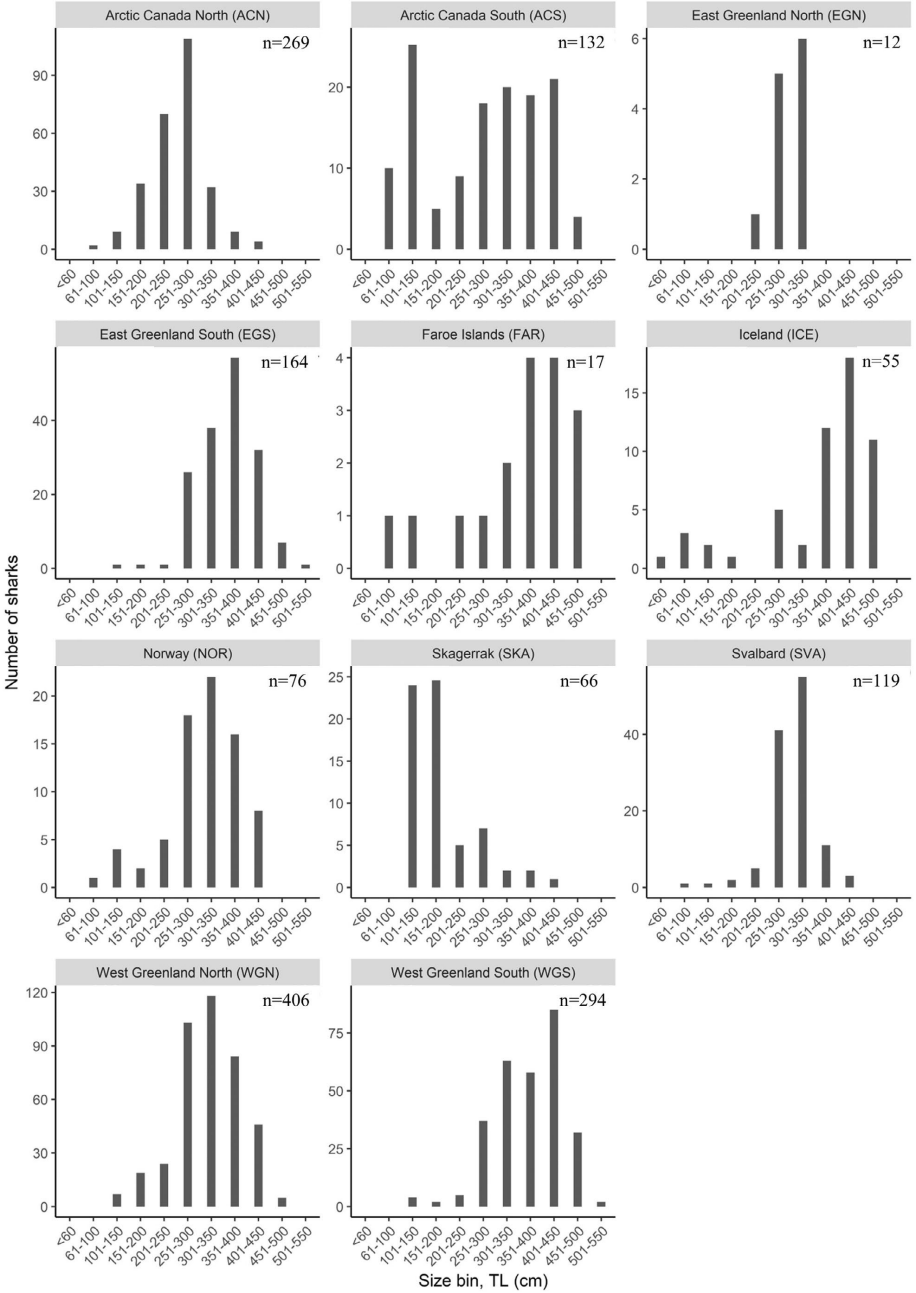
Greenland shark total length distributions (50 cm length bins) in each of the 11 analyzed regions.

**FIGURE 3:**
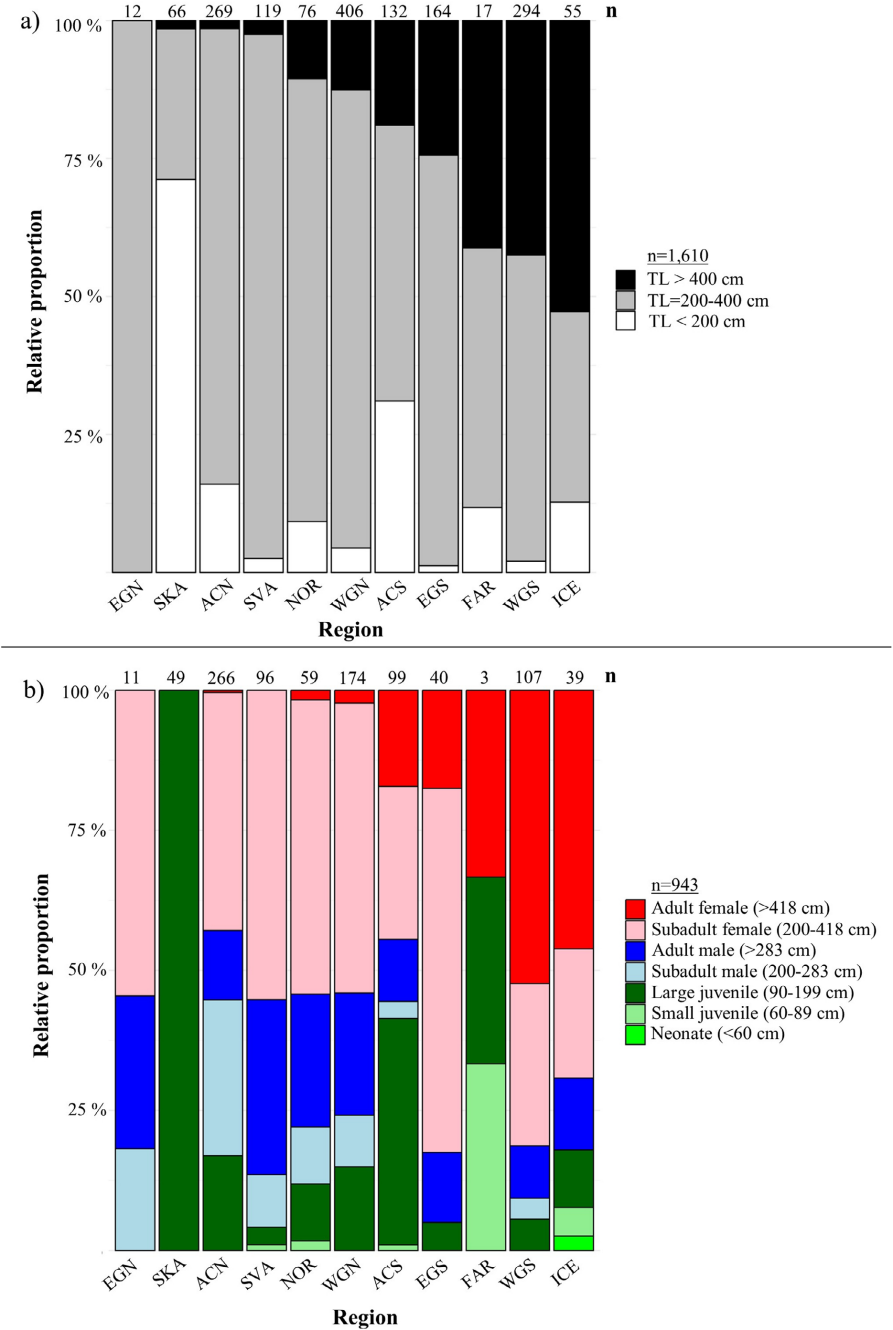
(a) Proportional occurrence of all analyzed sharks in three “Size groups” (total length, TL) TL < 200 cm, TL 200–400 cm, and TL > 400 cm, in each of the 11 geographical regions. (b) Proportional occurrence of all sharks assigned to one of the seven life stage categories (“Neonate”, “Small juvenile”, “Large juvenile”, “Subadult male”, “Adult male”, “Subadult female”, and “Adult female”). Regions are ordered based on proportion of the > 400 cm size class. For both panel a and b: ACN, Arctic Canada north; ACS, Arctic Canada south; EGN, East Greenland north; EGS, East Greenland south; FAR, Faroe Islands; ICE, Iceland; NOR, mainland Norway; SKA, Skagerrak; SVA, Svalbard; WGN, West Greenland north; WGS, West Greenland south.

The length composition differed significantly between regions with an average TL < 300 cm in ACN, ACS, EGN and SKA, an average TL between 300 and 350 cm in WGN, NOR, and SVA, and the largest sharks with an average TL > 350 cm in WGS, EGS, ICE, and FAR (Anderson‐Darling test, T.AD = 134.03, *n* = 1610, *p* < 0.001) (Figure 2). To ensure that the result is not skewed by including the two areas with considerably lower sample size (FAR and EGN), the statistical comparison was also done without these areas and the result was equally significant (*p* < 0.001). Length groups ‘Small’: TL < 200 cm, ‘Medium’: TL 200–400 cm, and ‘Large’: TL > 400 cm, for male and females combined, were unevenly distributed across the 11 geographic regions (χ^2^ = 627.6, df = 20, *n* = 1610, *p* < 0.001) (see contingency in Table S2). In general, “Medium” sharks dominated in all regions except for ICE and SKA, where “Large” and “Small” sharks dominated, respectively (Figure 3a). “Small” sharks were also abundant in ACS and ACN. “Large” sharks were most prevalent in ICE, WGS, and FAR, while they were rare in EGN, SKA, ACN, and SVA (Figure 3a).

For all regions, females occurred more frequently than males (Table 3) and “Subadult female” was the most common life stage in most regions, except in WGS and ICE, where “Adult female” was most common (Figure 3b). In contrast, “Adult female” was absent, or nearly so, in EGN, SKA, ACN, SVA, NOR, and WGN. “Adult male” was found in all regions and was generally more common than “Subadult male” except in ACN. Specimens classed as “Large juvenile” were not caught in EGN, but this could be due to limited sample size (*n* = 12) (Table 2). “Large juvenile”’ was present in all other regions and had the highest occurrence in SKA, ACS, and ACN (Figure 3b). Only six specimens were registered as “Small juvenile,” with a single specimen in each of four regions (ACS, FAR, NOR, and SVA) and two specimens in ICE, which is also the region where the only “Neonate” was recorded among the 1610 analyzed sharks (Figures 1a and 3b).

### 3.3 Depth and Temperature

**FIGURE 4:**
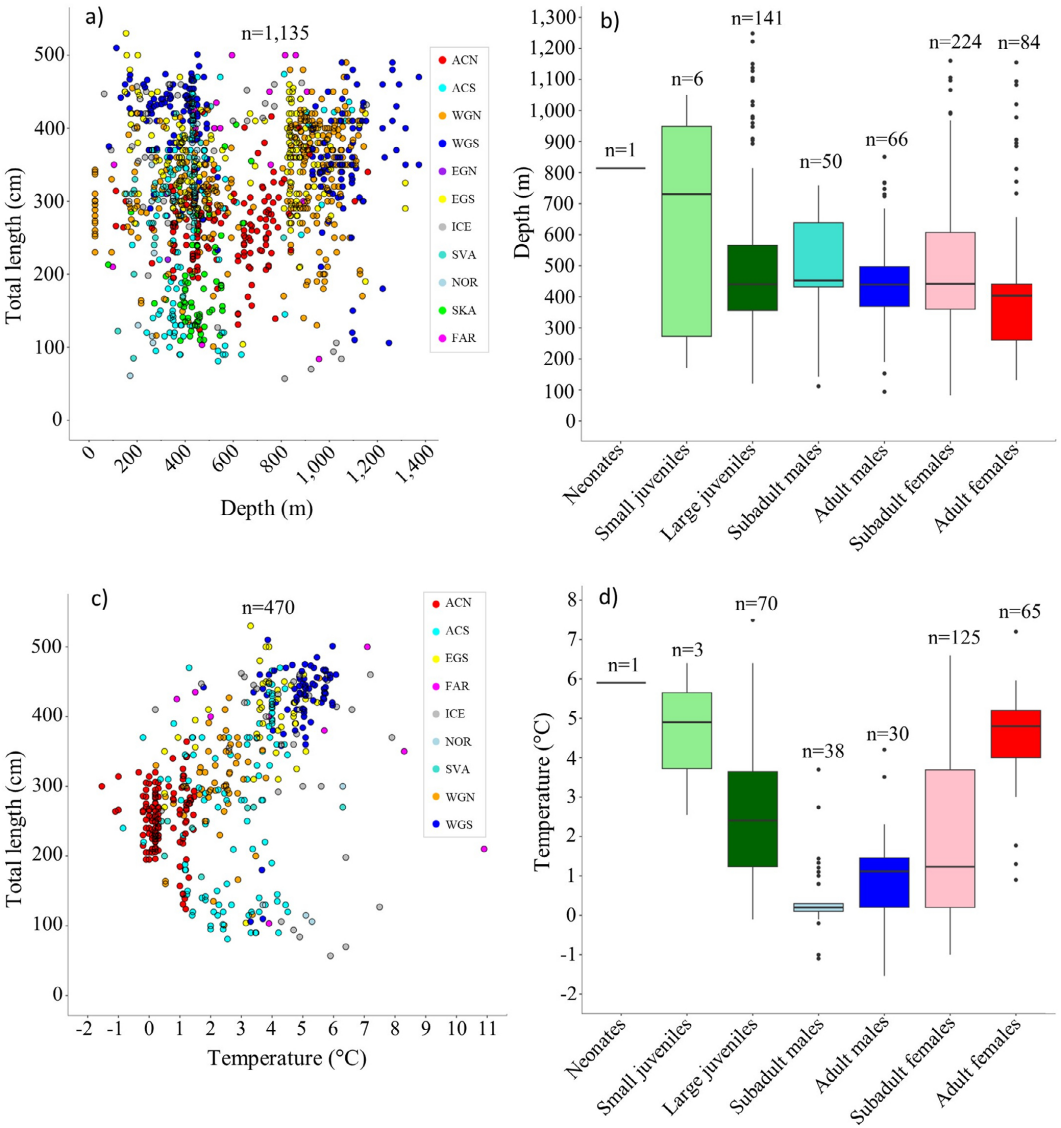
(a) Capture depth against total length (TL) across regions. ACN, Arctic Canada north; ACS, Arctic Canada south; EGN, East Greenland north; EGS, East Greenland south; FAR, Faroe Islands; ICE, Iceland; NOR, mainland Norway; SKA, Skagerrak; SVA, Svalbard; WGN, West Greenland north; WGS, West Greenland south. (b) Capture depth against life stage pooled for all regions. (c) Water temperature against TL across regions. (d) Water temperature against life stage pooled for all regions. For box plots in panel (b) and (d), whiskers represent maximum and minimum values that are not outliers, which are represented by filled dots. The box represents 1st and 3rd quartile and the line in the box represents the median.

Capture depth ranged from 25 m to 1375 m (*n* = 1135) and water temperatures from −1.54°C to −10.9°C (*n* = 470). There was an overall positive correlation between body size and capture depth (Linear regression, *F*_1,1113_ = 33.82, *n* = 1135, *p* < 0.001, *R* ^2^ = 0.019) but while the regression was significant, there is limited explanatory power of depth for body size. Also, regions showed opposite and weak trends (*p* < 0.001) (Figure 4a). Noticeably, for the six data‐rich regions (ACS, ACN, WGN, WGS, EGS, and SVA, *n* > 100, Table 2), the trend for body length‐at‐depth was negative in WGS and EGS, and positive in ACN, ACS, WGN, and SVA (Figure S1). There was a slight tendency for subadult life stages to occupy deeper waters than adults (Figure 4b), but the differences were not significant (Tukey Post hoc tests, *n* = 572, *p* > 0.07).

In general, there was a positive correlation between body length and water temperature irrespective of region (Linear regression, *F*_1,468_ = 116.78, *n* = 470, *p* < 0.001, *R* ^2^ = 0.20) (Figure 4c) except for ICE and FAR (Figure S2). Each life stage occupied specific water temperature ranges (Two‐way ANOVA, *F*_5,317_ = 5.10, *n* = 470, *p* < 0.001, Figure 4d) suggesting water temperature selection may take place throughout ontogeny. That is, subadult females were caught at a wide range of water temperatures (mean ± SD: 1.95°C ± 1.87°C, interquartile range, IQR: 0.20°C–3.69°C), whereas subadult males (mean ± SD: 0.42°C ± 0.84°C; IQR: 0.10°C–0.55°C) and adult males (mean ± SD: 1.06°C ± 1.12°C; IQR: 0.21°C–1.46°C) were caught in colder waters and had a narrower water temperature range (Figure 4d). Notably, adult females were caught at significantly higher water temperatures (4.57°C ± 1.07°C) than adult males and both sex subadult life stages (Tukey Post hoc tests, *n* = 332, *p* < 0.001) (Figure 4d). The apparent preference for warmer water among the “Adult female” category was seen across many regions, although they were not necessarily present or dominating in all regions characterized by warm water. Neonates and small juveniles also occupied warm waters (> 4.5°C) similar to adult females, but the sample size for these early life stages was small (*n*_neonates_ = 1 and *n*_small juveniles_ = 3). Large juveniles, on the other hand, appeared more indifferent to water temperature since this life stage was encountered in both warm and cold waters (mean ± SD: 2.60°C ± 1.52°C, IQR: 1.25°C–3.68°C) (Figure 4d).

### 3.4 Inshore–Offshore

**FIGURE 5:**
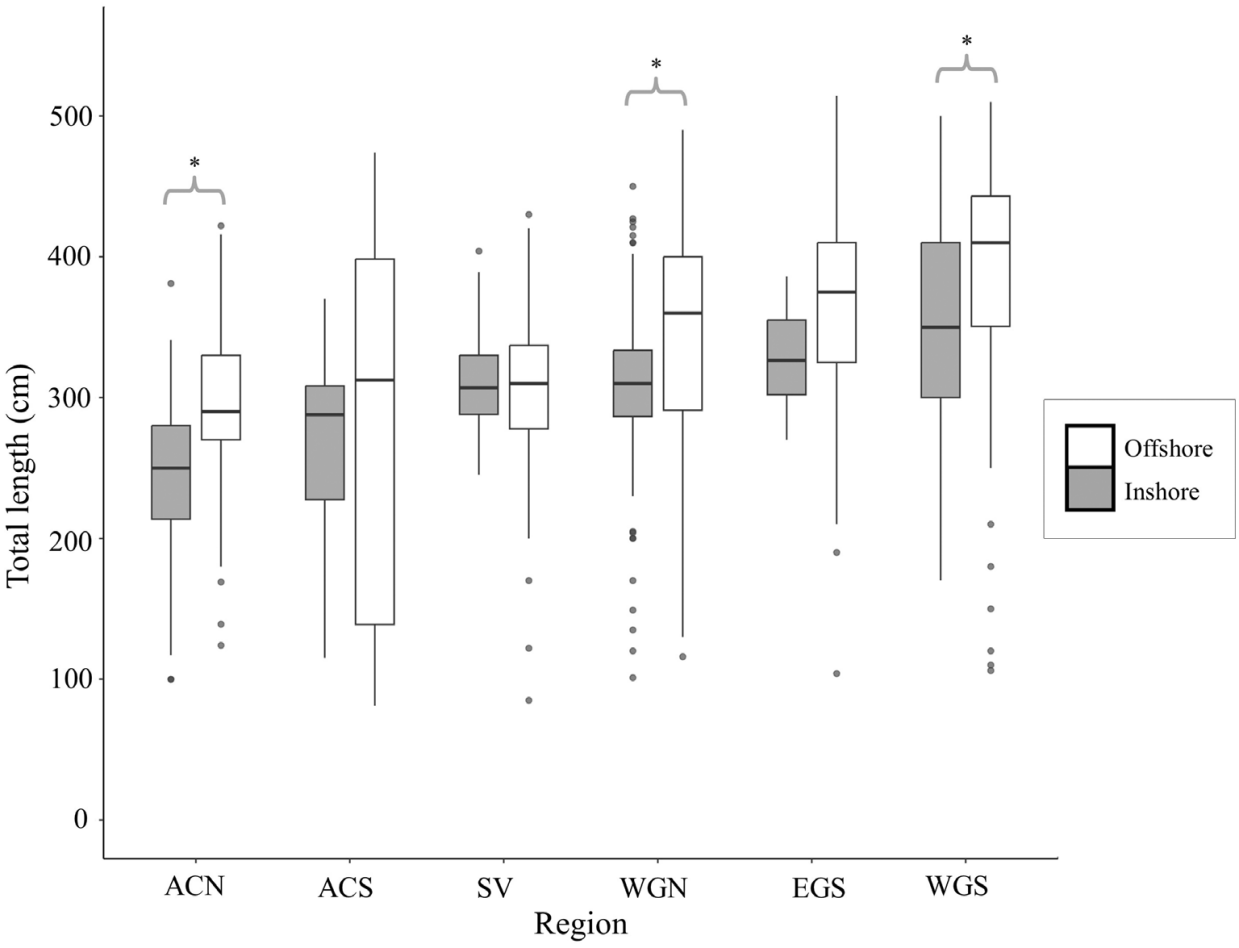
Boxplot of total length (TL) of sharks caught in offshore (red) versus inshore (blue) waters for data‐rich regions (*n* > 100) encompassing. ACN, Arctic Canada north; ACS, Arctic Canada south; EGS, East Greenland south; SVA, Svalbard; WGN, West Greenland north; WGS, West Greenland south. Significantly different body length composition within regions are marked with * (Kruskal Wallis test, *p* < 0.05). Whiskers represent maximum and minimum values that are not an outlier which are represented by filled gray dots. The box represents 1st and 3rd quartile and the line in the box represent the median.

Within the six data‐rich regions (*n* > 100), body sizes and sex ratios of “Inshore” versus “Offshore” areas were compared. Not all offshore sharks from the different regions were of similar size and life stage, but there was segregation in body size within the given regions. Hence, sharks from offshore waters were generally larger, especially in ACN, WGN, and WGS (Kruskal–Wallis, *H* = 89.2, *n* = 1384, *p* < 0.001, Figure 5). The sex ratios varied both between regions and between inshore and offshore waters (Table 3). In ACN and SVA, the sex ratio was similar in inshore and offshore waters, whereas for other regions females dominated in either inshore (WGN) or offshore (ACS) areas. In WGS and EGS, females dominated in both inshore and offshore waters. The offshore waters of ACS were notable as sharks here had the widest body size range (IQR: 139–398 cm) compared to the other five data‐rich regions (Figure 5). WGS was the only region where large sharks (TL > 400 cm) occurred frequently in both inshore (IQR: 300–410 cm) and offshore waters (IQR: 350–443 cm) (Figures 2 and 5).

### 3.5 Additional Greenland Sharks

**FIGURE 6:**
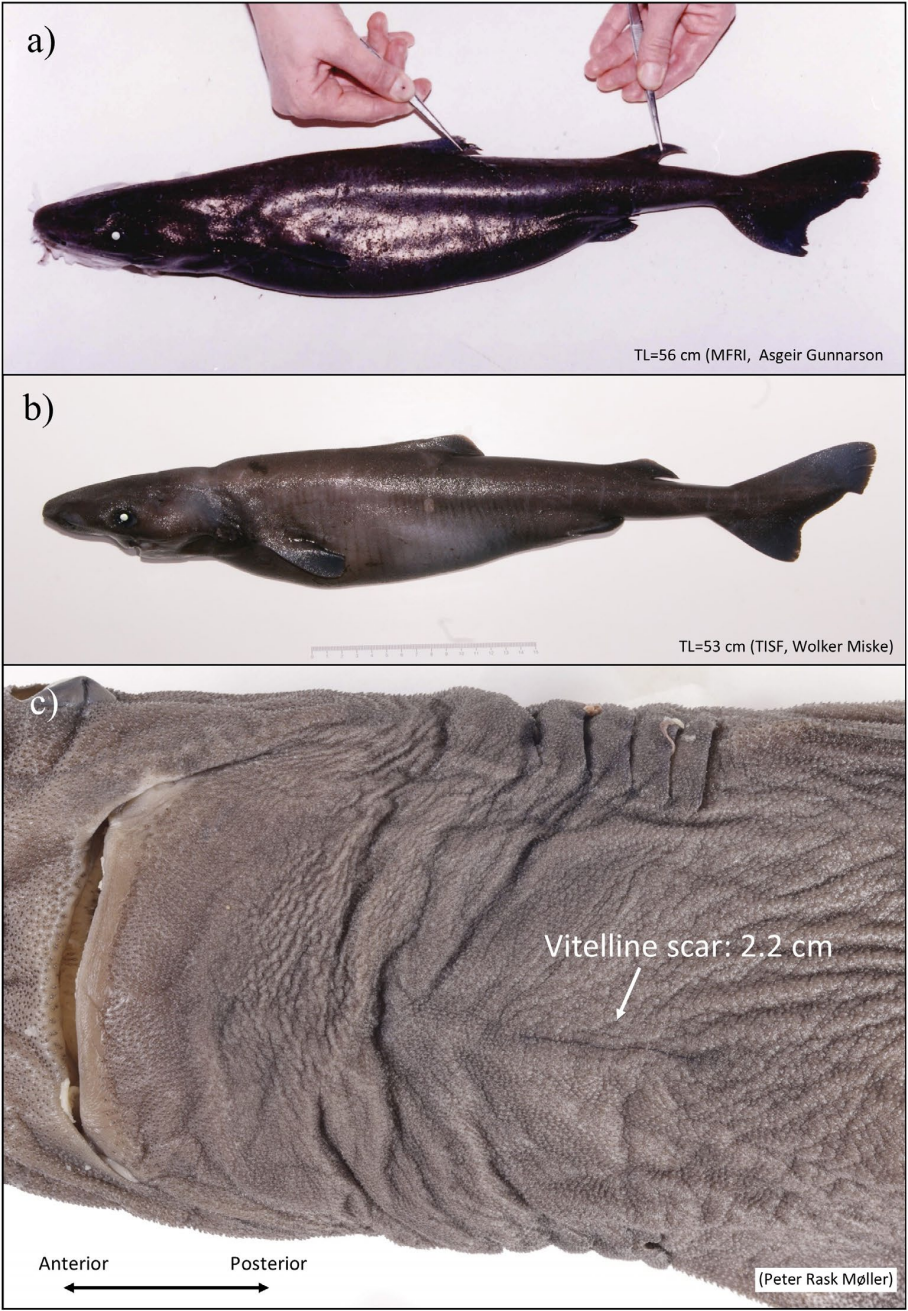
(a) Neonate Greenland shark from the Iceland Marine & Freshwater Research Institute (MFRI) database. (b, c) Neonate Greenland shark (id number: DMM IE/10000) with vitelline scar of 2.2 cm from the Thünen Institute of Sea Fisheries (TISF) database.

The 28 specimens from the Reykjanes Ridge and adjacent seas (*n* = 12), Barents Sea (*n* = 15), and southwest Greenland (*n* = 1) represent rare and exceptional occurrences of small‐sized sharks. The pelagically caught specimens near the Reykjanes Ridge were comprised of neonate specimens (*n* = 10, TL ~42–56 cm) and small juveniles (*n* = 2, TL 66–89 cm) (Table 5, Figure 1a). Six specimens were caught in scientific surveys and four in commercial fisheries, whereafter they were reported by the fisherman to MFRI due to their unusual small body size (Figure 6a,b). The pelagic gear operated at 400–700 m over bottom depths of 1300–2930 m and, notably, no larger sharks have been recorded pelagically in the Irminger Sea (cf. the databases of MFRI and TISF and Dolgov (2015)). The two neonates of Kukuev and Trunov (2002) (TL 41.8 and 46.7 cm) were caught in a pelagic gear east and west of the Reykjanes Ridge in 1981 and 1999, respectively. The sharks caught in the Barents Sea comprised small (*n* = 5, TL 80–82 cm) and large (*n* = 10, TL 92–115 cm) juveniles caught on long lines at depths of 205–285 m within a narrow geographic area in January 2022 (cf. University Museum of Bergen) (Figure 1a). One small juvenile (TL 65 cm) from southwest Greenland was caught in 1992 by RV Shinkai Maru at 1075 m depth (Table 5).

## 4 Discussion

The Squaliform order contains several species with well‐defined demographic segregations. For example, depth segregation by body size, sex, and maturity stage has been demonstrated for Portuguese dogfish *Centroscymnus coelolepis* , black dogfish *Centroscyllium fabricii* , and leafscale gulper shark *Centrophorus squamosus* (Moura et al. 2014; Yano 1995; Yano and Tanaka 1988). Despite the recent increase in studies on Greenland sharks, basic knowledge of potential segregation in relation to depth and water temperature for different life stages remains sparse. Furthermore, spatial relationships among key life history events and/or life stages (Sims 2006) are yet to be identified for Greenland sharks.

### 4.1 Temperature‐Depth Habitat Preferences

We found that depth had little importance in terms of sex or body size composition, which was surprising given many deep‐water fishes exhibit this type of relationship (Polloni et al. 1979; Macpherson and Duarte 1991). Also, a correlation between increasing size at increasing depth had previously been suggested by Yano et al. (2007) for Greenland sharks in West Greenland; however, this was for a limited sample (*n* = 49). Our study found no distinct correlation between depth and size or sex across regions. Instead, size correlated with temperature, indicating that some sizes of Greenland sharks choose depths or areas based on temperature preferences. The most distinct pattern was observed for the largest specimens (TL > 400 cm, *n* = 293) that typically were associated with warmer waters (> 4°C). We are confident that the largest sharks > 400 cm are all females, which are also potentially sexually mature, or soon to be sexually mature. This is based on several factors: (1) A range of published reports have identified a distinct sexual dimorphism in Greenland sharks, with females outgrowing males (e.g., Hansen 1963b; Yano et al. 2007; MacNeil et al. 2012; Nielsen 2018, this study); (2) Size‐at‐first maturity for females is TL ≥ 400 cm (Nielsen et al. 2020); (3) Males rarely exceed 350 cm and have a maximum reported TL of 375 cm (Nielsen et al. 2020). When considering life stage, this trend of the largest sharks in warmest waters was even clearer, as adult females (*n* = 105) were caught at an average water temperature of 4.7°C, while males and subadult females were typically caught below 3.5°C. Such a temperature trend is not surprising. Fish often seek higher water temperatures during sexual maturation (e.g., Bezerra et al. 2013; de Mendonca et al. 2020; Mull and Lowe 2010), a behavior that also has been documented for several shark species (e.g., Bangley et al. 2018; Pratt et al. 2022). This preference is linked to the metabolic processes associated with increased body and liver mass, vitellogenesis, and subsequent fetal development during gestation, which occur more rapidly for ectotherms at higher temperatures (Christiansen et al. 1997; Sims 2006; Sulikowski et al. 2016). Therefore, the occurrence of large adult female Greenland sharks in regions influenced by warm Atlantic water is not per se surprising and presumably linked to their reproductive cycle. The full reproductive cycle of male and female Greenland sharks is described in Nielsen et al. (2020).

The warm‐water preference of large and potentially sexually mature females (TL > 400 cm) is an important finding of the current study, which can be further scrutinized at the regional level. In southeast Greenland, southern Arctic Canada, and in the southern parts of northwest Greenland, the trend was especially evident, as females > 400 cm were predominant in warmer, “Atlantic” offshore waters, while being scarce or absent in adjacent colder inshore waters (Figure 5) (see Born and Böcher 1999; Gjelstrup et al. 2022; Mortensen et al. 2011, 2018, 2022; Straneo et al. 2010 for descriptions of oceanographic conditions in the relevant areas). These large females also appear rare or absent in the cold fjords of northwest Greenland, and in both inshore and offshore waters of northern Arctic Canada. Historical data from both northwest and southeast Greenland also support a distributional pattern of > 400 cm females not preferring colder water masses. In more detail, unpublished observations made by the Norwegian marine biologist Bjørn Berland, who collected information on maturity status for hundreds of Greenland sharks from 1959 to 1960 (during a targeted shark liver fishery), specifically mentioned that sexually mature females were only caught in offshore waters of southeast Greenland, whereas the smaller immature specimens were encountered in inshore waters of the Umivik Fjord (64° N), which is a cold water fjord (Berland unpublished data examined by JSC and JN; see also Nielsen et al. 2020, Figure 1). Also, from northwest Greenland, Hansen (1963b) reported lengths of 156 sharks to be TL 125–374 cm, collected in 1936 from inshore waters in Disko Bay (69° N) and Uummannaq Fjord (71° N). Based on their size, none of these were large enough to be potentially mature females. This does not mean that we suggest that large (TL > 400 cm) females can not be present in these particalar places. The point is, that for these particular places the historical litterature also supports a difference in size between inshore and offshore waters. The only region where TL > 400 cm females have been commonly encountered in both inshore and offshore waters was southwest Greenland, which is also characterized by a milder Atlantic climate. Therefore, the presence of large females TL > 400 cm throughout this region aligns with our overall expectation that the adult females prefer warm water (although they may occupy cold water masses for short time periods in inshore waters of southwest Greenland, see Nielsen 2018). For northeast Greenland, Iceland, and the Faroe Islands, data is scarce, but the trends appeared similar. Specifically, no large females are known from the cold waters of northeast Greenland (Gjelstrup et al. 2022), while our study shows that large females were relatively more common in the warmer waters of Iceland and the Faroes Islands. This warm water preference of TL > 400 cm females is also supported by 3–13 months tagging studies with temperature loggers in the Northwest Atlantic (Campana et al. 2015; Nielsen 2018).

Large and potentially mature females (TL > 400 cm) were, however, not reported in all warm water regions in the northeast Atlantic; wherefore suitable water temperature does not necessarily imply their presence. Among 65 sharks from Kongsfjorden (78° N, Svalbard) (see Fisk et al. 2012; Leclerc et al. 2012) and 66 sharks from Skagerrak (the present study), only four females were large enough to be potentially mature (i.e., TL > 400 cm) despite both Skagerrak and Svalbard being regions influenced by warm Atlantic waters (Furnes et al. 1986; Sætre 2007). Also, for the Norway mainland, ten out of 76 Greenland sharks were > 400 cm females, revealing rather few of these potentially adult females despite very suitable water temperatures being present throughout the region. However, a somewhat low occurrence of large females in Norway aligns with Rusyaev and Orlov (2013), who did not report any potentially mature females out of 75 specimens from across the Barents Sea (the Russian and the Norwegian part). Of the ten large females in Norway, nine were caught in Vågsfjorden (68° N, data not shown). Overall, for Skagerrak, Svalbard, and the Norway mainland, nonthermal factors may be contributing to the distribution of life stages in these areas of the Northeast Atlantic. It is also possible that the sample size in some regions may be too small or spatially inadequate, such that factors that potentially are shaping distribution patterns are not evident in the current analyses.

### 4.2 Mating Areas

Data on the maturation status of individual sharks were not available, wherefore specific mating areas could not be identified. Adult males and adult females showing resident and overlapping behavior in time and space in Vågsfjorden, Norway, have been documented from tagging efforts (J. Nielsen, unpublished data). However, only in southwest Greenland have males with sperm in the seminal vesicles and extruded clasper spurs been reported, and exclusively from inshore areas. Females with ripe ova have been observed in nearby offshore waters of southwest Greenland (see Nielsen et al. 2020) making this the only region where such male–female observations align. Yet, the currently available data are too sparse to evaluate whether mating activity occurs here. Clearly, more data on maturation status are needed, and we encourage scientific monitoring programs to extend sampling protocols to include anatomical assessment of maturation status for specimens unable to survive if released. Protocols to assess maturation stage of Greenland shark males and females are available in Nielsen et al. (2020).

### 4.3 Pupping Grounds

More than sixty years ago, Hansen (1963b) suggested that pupping grounds in Greenland were located offshore in deep waters beyond the reach of commercial fisheries. This is still a valid hypothesis considering the overall rarity of gravid females and neonates (TL < 60 cm). In fact, only a single gravid female and two free‐swimming neonates are known from the scientific literature (see Koefoed 1957; Kukuev and Trunov 2002). The female carrying near‐term pups (TL 37 cm) was caught in offshore Faroese Islands shelf waters during a targeted Greenland shark fishery in 1954 (Carlson 1958; Koefoed 1957). However, the Faroese shelf is not likely a pupping ground for Greenland sharks because neither gravid females nor neonates have been reported from this area despite high fishing activity. Among the 1610 sharks analyzed in this study, the only neonate specimen (TL 57 cm) was caught in a bottom trawl on the shelf south of Iceland (depth 819 m, red star in Figure 1)—a location close to the Reykjanes Ridge. This abyssal mountain ridge is particularly interesting as the two smallest free‐swimming Greenland sharks (TL 41.8 cm and TL 46.7 cm, Kukuev and Trunov 2002) were caught in bathypelagic waters on the eastern and western side of the ridge in 1981 and 1999, respectively (Table 5). Furthermore, in the Icelandic (MFRI), Russian and German (TISF) pelagic surveys for beaked redfish that include depths down to 1000 m (ICES 2022a), eight neonates have been caught on or close to the Reykjanes Ridge (cf. the additional sharks, Table 5). One neonate specimen (TL 53 cm) is preserved in the collection of the German Oceanographic Museum (id number: DMM IE/10000) and a visual inspection by JN and PRM revealed a vitelline scar in accordance with the early neonate stage (Figure 6c). It is noteworthy that a comparable pelagic redfish survey along the Knipovich Ridge in the Norwegian Sea (2009–2022, ICES 2022a) has not caught Greenland sharks (pers. comm. Hannes Höffle from IMR). Similarly, no Greenland sharks have been reported from other pelagic surveys in Greenland, Iceland, Norway, and Faroe Islands targeting mackerel *Scomber scombrus* , capelin *Mallotus villosus* , or blue whiting *Micromesistius poutassou* . Also, the source databases for our study contain more than 55,000 bottom trawl hauls (data not shown), which also sample pelagic fauna. Therefore, we conclude that neonates are not present in shelf waters of Greenland, Canada, Norway, or Svalbard, and their unique occurrence seems limited to areas near the Reykjanes Ridge and in the Irminger Sea. Greenland sharks have been documented from depths of 2.9 km, and we suggest that pupping sites may be located in the bathyal waters on or in the vicinity of the Reykjanes Ridge, along the slopes or even deeper in the Icelandic Basin or Irminger Basin (Figure 1a). The Russian Federal Research Institute of Fisheries and Oceanography (PINRO) has also conducted extensive surveys at the Reykjanes Ridge and in the Irminger Sea (ICES 2022a, 2022b). However, except for the single neonate described in Dolgov (2015) data from the Russian survey are presently unavailable.

### 4.4 Nursery Areas

Similar to neonates, records of small juveniles (TL 60–89 cm) are scarce among the 1610 sharks analyzed in our study. The few observations are widespread, with one specimen each in Arctic Canada south, mainland Norway, Svalbard, and Faroe Islands, and two specimens from Iceland. A single small juvenile from southwest Greenland has also been identified in a museum collection. The two Icelandic specimens were caught in bottom trawls at 924 m and 1050 m near the Reykjanes Ridge, which, when considered along with the information from Table 5 on two other juveniles and several neonate specimens, indicates that the waters south of Iceland near the Reykjanes Ridge and/or the Irminger Sea are potentially important nursery habitats.

Five small juveniles and ten large juveniles (TL 90–200 cm) have also been identified from the same location in the southeastern Barents Sea (Table 5 and Figure 1), suggesting another potential nursery area not previously identified. The only previously suggested nursery ground was based on the presence of large juveniles in Scott Inlet/Sam Ford Trough on Baffin Island (ACN) (Hussey et al. 2015). Our study found that “small” sharks were abundant in Arctic Canada south and north, which supports and expands on the findings by Hussey et al. (2015) concerning the potential for nursery areas in this region of the Northwest Atlantic. Our analysis indicates that Skagerrak has a disproportionately high occurrence of large juveniles and is not commonly occupied by adult Greenland sharks. This finding is remarkable, as this particular life stage was previously only associated with either high Arctic or deep‐sea waters of the continental slopes (Hussey et al. 2015; Nielsen et al. 2014). Historical records available from Denmark that include information on strandings and rare bycatches over the past 150 years help to support our observation of large juveniles in Skagerrak (Nielsen et al. 2019). However, it is important to emphasize that only the deepest parts of Skagerrak (> 400 m), which connect to the continental shelf break, should be considered suitable habitat for Greenland sharks, and not the much shallower inner waters of Denmark and Sweden. Hence, we consider Skagerrak as a potential nursery area for Greenland sharks.

For all suggested nursery areas highlighted here, it should be emphasized that they do not necessarily fulfill all three criteria for shark nursery areas as suggested by Heupel et al. (2007): (1) sharks more commonly encountered in the area than in other areas; (2) sharks have a tendency to remain or return for extended periods; and (3) the area or habitat is repeatedly used across years. Nonetheless, the identified areas should be considered important, and we encourage further research and improved monitoring efforts to explore these areas further.

## 5 Conclusion and Recommendations

The Greenland shark exhibits some demographic segregation across the northern North Atlantic, which is not surprising and has widely been documented among other sharks and bony fishes (e.g., Harden Jones 1968). The most distinct findings on Greenland sharks of the current study are (1) regions characterized by cold Arctic water masses are typically dominated by males and subadult females; (2) adult females reside in regions and areas with warmer Atlantic water masses, although warmer waters do not necessarily infer the presence of adult females, cf. the Northeast Atlantic regions; (3) large juveniles are common both in high‐Arctic and temperate areas (e.g., Skagerrak); (4) the remarkable absence of neonates in the evaluated data and the scientific literature in general (except two specimens in Rusyaev and Orlov 2013) strongly suggests that Greenland sharks are neither born in shelf waters nor in Arctic fjords. Our findings suggest that pupping occurs in the bathypelagic or benthic habitats of the mid‐Atlantic Ridge and/or the Irminger Sea, which are the only areas where neonates (TL < 60 cm) have been documented.

The findings on the whereabouts and life history of Greenland sharks allow for more precise conservation measures to be enforced. The longevity of Greenland sharks is exceptional (Nielsen et al. 2016), and we consider regional protection to be of great importance to counteract the present Red List status (Vulnerable). We argue that especially the large and potentially mature females (TL > 400 cm) should be safeguarded in inshore and offshore waters of southwest Greenland (WGS), the offshore waters of southern Arctic Canada (ACS), southeast Greenland (SEG), and Iceland (ICE) although the current dataset is somewhat limited from Icelandic waters. Nonetheless, for these four regions, bycatch of Greenland sharks in commercial fisheries should be avoided or strongly restricted, and the release of bycatch sharks should be ensured with nonlethal methods. A caveat for the findings of the current study is that most data are obtained within a limited time slot from late spring to early autumn (data not shown). Hence, spatial shift with season is relevant for future studies. We recommend that potential seasonality in spatial distribution is scrutinized further within all analyzed regions by using, for example, satellite tracking and environmental DNA technologies. Also, we encourage the standardization of sampling protocols across scientific institutions in Canada, Greenland, Iceland, Faroe Islands, and Norway so that catch‐per‐unit‐effort data becomes available. Over time, improved data collection will also provide larger and more homogenous datasets in terms of available covariables (depth, water temperature, and sex). Such data will allow for detailed correlation models potentially to reveal further trends and patterns than described in this study. Correlation models were investigated on the current data but not found informative given the skewness of data between and within regions (data not shown). For the entire northeast Atlantic, from Iceland to the Barents Sea, more data on body size, depth, and water temperature are especially warranted, as well as data from all evaluated regions. Therefore, scientific activities are encouraged to routinely record length and sex of captured Greenland sharks, and for specimens that are dissected, the reproductive status should be classified according to the species‐specific maturation scale proposed by Nielsen et al. (2020).

## Author Contributions

**Julius Nielsen:** conceptualization (lead), data curation (equal), formal analysis (equal), investigation (lead), methodology (equal), project administration (lead), writing – original draft (equal), writing – review and editing (equal). **Jørgen Schou Christiansen:** conceptualization (equal), formal analysis (equal), investigation (equal), methodology (equal), supervision (equal), validation (equal), visualization (equal), writing – original draft (equal), writing – review and editing (equal). **Kim Præbel:** formal analysis (supporting), investigation (supporting), methodology (supporting), validation (supporting), writing – review and editing (supporting). **Peter Rask Møller:** data curation (supporting), formal analysis (supporting), investigation (supporting), supervision (supporting), visualization (supporting), writing – review and editing (supporting). **Brynn Devine:** conceptualization (supporting), data curation (supporting), investigation (equal), methodology (supporting), resources (supporting), visualization (supporting), writing – review and editing (supporting). **Klara Jakobsdottir:** data curation (supporting), investigation (supporting), resources (supporting), visualization (supporting), writing – review and editing (supporting). **Nicolas Straube:** data curation (equal), investigation (supporting), visualization (supporting), writing – review and editing (supporting). **Adriana Nogueira:** data curation (supporting), visualization (supporting), writing – review and editing (supporting). **Margaret Treble:** data curation (supporting), validation (supporting), visualization (supporting), writing – review and editing (supporting). **Kevin Hedges:** data curation (supporting), visualization (supporting), writing – review and editing (supporting). **Sheila Atchison:** data curation (supporting), visualization (supporting), writing – review and editing (supporting). **Lise Helen Ofstad:** data curation (supporting), visualization (supporting), writing – review and editing (supporting). **Claudia Junge:** data curation (supporting), visualization (supporting), writing – review and editing (supporting). **Laura Wheeland:** data curation (supporting), visualization (supporting), writing – review and editing (supporting). **Rasmus Hedeholm:** conceptualization (equal), data curation (equal), formal analysis (equal), investigation (equal), methodology (equal), resources (equal), software (lead), supervision (equal), validation (equal), visualization (equal), writing – original draft (equal), writing – review and editing (equal).

## Conflicts of Interest

The authors declare no conflicts of interest.

## Supporting information


Appendix S1.

Appendix S2.

Figure S1.

Table S1.

Table S2.

## Data Availability

All relevant data are contained in the manuscript's Table S1.

## References

[ece371564-bib-0001] Bangley, C. W. , L. Paramore , D. S. Shiffman , and R. A. Rulifson . 2018. “Increased Abundance and Nursery Habitat Use of the Bull Shark ( *Carcharhinus leucas* ) in Response to a Changing Environment in a Warm‐Temperate Estuary.” Scientific Reports 6: 6018. 10.1038/s41598-018-24510-z. PMC5902476 29662126

[ece371564-bib-0002] Bezerra, N. P. A. , C. A. F. Fernandes , F. V. Albuquerque , V. Pedrosa , F. Hazin , and P. Travassos . 2013. “Reproduction of Blackfin Tuna, *Thunnus atlanticus* (Perciformes: Scombridae) in the Saint Peter and Saint Paul Archipelago, Equatorial Atlantic, Brazil.” Revista de Biología Tropical 61, no. 3: 1327–1339. 24027926

[ece371564-bib-0003] Born, E. W. , and J. Böcher . 1999. Grønlands Økologi—en grundbog. Atuakkiorfik.

[ece371564-bib-0004] Campana, S. E. , A. T. Fisk , and A. P. Klimley . 2015. “Movements of Arctic and Northwest Atlantic Greenland Sharks ( *Somniosus microcephalus* ) Monitored With Archival Satellite Pop‐Up Tags Suggest Long‐Range Migrations.” Deep‐Sea Research Part II 115: 109–115. 10.1016/j.dsr2.2013.11.001.

[ece371564-bib-0005] Carlson, L. 1958. “Håkjerringa og håkjerringfisket: Vol. IV, No. 1.” In Fiskeridirektoratets Skrifter (in Norwegian) [In Norwegian.]. Director of Fisheries.

[ece371564-bib-0006] Christiansen, J. S. , H. Schurmann , and L. Karamushko . 1997. “Thermal Behaviour of Polar Fish: A Brief Survey and Suggestions for Research.” Cybium 21, no. 4: 353–362.

[ece371564-bib-0007] Davis, B. , D. L. VanderZwaag , A. Cosandey‐Godin , N. E. Hussey , S. T. Kessel , and B. Worm . 2013. “The Conservation of the Greenland Shark (*Somniosus microcephalus*): Setting Scientific, Law, and Policy Coordinates for Avoiding a Species at Risk.” Journal of International Wildlife Law & Policy 16, no. 300: 300–330. 10.1080/13880292.2013.805073.

[ece371564-bib-0075] Devine, B. M. , L. J. Wheeland , and J. A. D. Fisher . 2018. “First estimates of Greenland shark (*Somniosus microcephalus*) local abundances in Arctic waters.” Scientific Reports 8, no. 974: 1–10. 10.1038/s41598-017-19115-x. 29343730 PMC5772532

[ece371564-bib-0008] de Mendonca, S. A. , B. C. L. Macena , C. B. B. de Araujo , N. P. A. Bezerra , and F. H. V. Hazin . 2020. “Dancing With the Devil: Courtship Behaviour, Mating Evidences and Population Structure of the *Mobula tarapacana* (Myliobatiformes: Mobulidae) in a Remote Archipelago in the Equatorial Mid‐Atlantic Ocean.” Neotropical Ichthyology 18, no. 3. 10.1590/1982-0224-2020-0008.

[ece371564-bib-0009] Dolgov, A. V . 2015. “Composition and Structure of the Mesopelagic Fish Communities in the Irminger Sea and Adjacent Waters.” Journal of Ichthyology 55, no. 1: 53–68.

[ece371564-bib-0010] Doubleday, W. G. 1981. “Manual on Groundfish Surveys in the Northwest Atlantic.” NAFO Scientific Council Studies 2: 7–55.

[ece371564-bib-0011] Edwards, J. E. , K. J. Hedges , and N. E. Hussey . 2021. “Seasonal Residency, Activity Space, and Use of Deep‐Water Channels by Greenland Sharks (*Somniosus microcephalus*) in an Arctic Fjord System.” Canadian Journal of Fisheries and Aquatic Sciences 79, no. 2. 10.1139/cjfas-2021-0009.

[ece371564-bib-0012] Edwards, J. E. , E. Hiltz , F. Broell , et al. 2019. “Advancing Research for the Management of Long‐Lived Species: A Case Study on the Greenland Shark.” Frontiers in Marine Science 6, no. 87. 10.3389/fmars.2019.00087.

[ece371564-bib-0074] Edwards, J. E. , J. E. Edwards , K. J. Hedges , S. T. Kessel , and N. E. Hussey . 2022. “Multi‐Year Acoustic Tracking Reveals Transient Movements, Recurring Hotspots, and Apparent Seasonality in the Coastal‐Offshore Presence of Greenland Sharks (*Somniosus microcephalus*).” Frontiers in Marine Science 9: 902854. 10.3389/fmars.2022.902854.

[ece371564-bib-0013] Fisk, A. T. , C. Lydersen , and K. M. Kovacs . 2012. “Archival Pop‐Off Tag Tracking of Greenland Sharks *Somniosus microcephalus* in the High Arctic Waters of Svalbard, Norway.” Marine Ecology Progress Series 468: 255–265. 10.3354/meps09962.

[ece371564-bib-0014] Fulton, S. , W. Walkusz , S. Atchison , and F. Cyr . 2024. “Information to Support the Assessment of Northern Shrimp, *Pandalus borealis*, and Striped Shrimp, *Pandalus montagui*, in the Eastern and Western Assessment Zones (2024/016; IV).”

[ece371564-bib-0015] Furnes, G. K. , B. Hackett , and R. Sætre . 1986. “Retroflection of Atlantic Water in the Norwegian Trench.” Deep‐Sea Research 33, no. 2: 247–265.

[ece371564-bib-0016] Gjelstrup, C. V. B. , M. K. Sejr , L. De Steur , et al. 2022. “Vertical Redistribution of Principle Water Masses on the Northeast Greenland Shelf.” Nature Communications: 1–12. 10.1038/s41467-022-35413-z. PMC9741604 36496498

[ece371564-bib-0018] Hansen, P. M. 1963a. “Tagging Experiments With the Greenland Shark (*Somniosus microcephalus* (Bloch and Schneider)) in Subarea 1.” International Commission for the Northwest Atlantic Fisheries: 172–175, Special Publication No. 4.

[ece371564-bib-0017] Hansen, P. M. 1963b. “Hajer i Grønlandske farvande.” In Danmarks Fiskeri‐og Havundersøgelser [In Danish.]. Danmarks Fiskerir‐ og Havundersrøgelser.

[ece371564-bib-0019] Harden Jones, F. R. 1968. Fish Migration. Edward Arnold.

[ece371564-bib-0020] Hedges, K. 2023. “Report on Greenland Halibut (*Reinhardtius hippoglossoides*) Caught During the 2022 Trawl Survey in Subarea 0 (Serial No. N7418; NAFO SCR Doc 23/029).”

[ece371564-bib-0021] Heupel, M. R. , J. K. Carlson , and C. A. Simpfendorfer . 2007. “Shark Nursery Areas: Concepts, Definition, Characterization and Assumptions.” Marine Ecology Progress Series 337: 287–297.

[ece371564-bib-0022] Hussey, N. E. , C.‐G. Aurelie , R. P. Walter , K. J. Hedges , S. T. Kessel , and A. T. Fisk . 2015. “Juvenile Greenland Sharks *Somniosus microcephalus* (Bloch & Schneider, 1801) in the Canadian Arctic.” Polar Biology 38: 493–504. 10.1007/s00300-014-1610-y.

[ece371564-bib-0076] Hussey, N. E. , K. J. Hedges , and A. Barkley . 2018. “Mark Report Satellite Tags (mrPATs) to Detail Large‐Scale Horizontal Movements of Deep Water Species: First Results for the Greenland Shark (*Somniosus microcephalus*).” Deep‐Sea Research Part I 134: 32–40. 10.1016/j.dsr.2018.03.002.

[ece371564-bib-0023] ICES . 2015. “Manual for the International Deep Pelagic Ecosystem Survey in the Irminger Sea and Adjacent Waters. Series of ICES Survey Protocols SISP 11—IDEEPS VI.” 10.17895/ices.pub.7584.

[ece371564-bib-0025] ICES . 2021a. “Working Group on Elasmobranch Fishes (WGEF).” In ICES Scientific Reports, vol. 3, Issue 59. 10.17895/ices.pub.8199.

[ece371564-bib-0024] ICES . 2021b. “Northwestern Working Group (NWWG).” In ICES, vol. 838. 10.17895/ices.pub.8186.

[ece371564-bib-0026] ICES . 2022a. “Working Group on International Deep Pelagic Ecosystem Surveys (WGIDEEPS).” ICES Scientific Reports. 10.17895/ices.pub.20401581.v1.

[ece371564-bib-0027] ICES . 2022b. “Working Group on the Biology and Assessment of Deep‐Sea Fisheries Resources (WGDEEP).” ICES Scientific Reports. ICES Scientific Reports. 10.17895/ices.pub.20037233.v1.

[ece371564-bib-0029] ICES . 2023a. “Stock Annex: Cod (*Gadus morhua*) in ICES Subarea 14 and NAFO Division 1.F (East Greenland, South Greenland).” ICES Stock Annexes 31. 10.17895/ices.pub.23742135.

[ece371564-bib-0028] ICES . 2023b. “Definition and Rationale for ICES Ecoregions.” General ICES Advice Guidelines. 10.17895/ices.advice.23634480.v1.

[ece371564-bib-0030] Jakobsdóttir, K. B. , E. Hjörleifsson , H. Björnsson , J. Sólmundsson , K. Kristinsson , and V. Bogason . 2023. “Handbók um stofnmælingu botnfiska að haustlagi 2023 (Kver Hafrannsóknastofnunar. KV 2023–5).” [In Icelandic.]. https://www.hafogvatn.is/static/research/files/1695381391‐handbok_smh_2023.pdf.

[ece371564-bib-0031] Jónsdóttir, I. G. , H. Karlsson , H. Björnsson , J. Sólmundsson , K. B. Jakobsdóttir , and V. Bogason . 2024. “Handbók um stofnmælingu botnfiska á Íslandsmiðum 2024 (Hafrannsóknastofnun, Kver, KV 2024–01).” [In Icelandic.]. https://www.hafogvatn.is/static/research/files/kv2024_01.pdf.

[ece371564-bib-0032] Jørgensen, O. A. 2011. “Bottom Trawl Survey in Baffin Bay, NAFO Divisions 1A, 2010. (NAFO Scientific Council Research Document 11/10).”

[ece371564-bib-0033] Koefoed, E. 1957. “2. A Uterine Foetus and the Uterus From a Greenland Shark.” In Notes on the Greenland Shark, 8–12. Fiskeridirektoratets Skrifter.

[ece371564-bib-0034] Kukuev, E. I. , and I. A. Trunov . 2002. “The Composition of Ichthyofauna of the Meso‐ and Bathypelagic Zones of the Irminger Current and of Adjacent Waters.” Journal of Ichthyology 42: 377–384.

[ece371564-bib-0035] Kulka, D. W. , C. F. Cotton , B. Andersen , D. Derrick , K. Herman , and N. K. Dulvy . 2020. “Somniosus Microcephalus.” The IUCN Red List of Threatened Species 2020, e.T60213A1.

[ece371564-bib-0036] Leclerc, L. M. E. , C. Lydersen , T. Haug , L. Bachmann , A. T. Fisk , and K. M. Kovacs . 2012. “A Missing Piece in the Arctic Food Web Puzzle? Stomach Contents of Greenland Sharks Sampled in Svalbard, Norway.” Polar Biology 35, no. 8: 1197–1208. 10.1007/s00300-012-1166-7.

[ece371564-bib-0037] MacNeil, M. A. , B. C. McMeans , N. E. Hussey , et al. 2012. “Biology of the Greenland Shark *Somniosus microcephalus* .” Journal of Fish Biology 80: 991–1018. 10.1111/j.1095-8649.2012.03257.x. 22497371

[ece371564-bib-0038] Macpherson, E. , and C. M. Duarte . 1991. “Bathymetric Trends in Demersal Fish Size: Is There a General Relationship?” Marine Ecology Progress Series 71, no. 2: 103–112.

[ece371564-bib-0039] McMeans, B. C. , J. Svavarsson , S. Dennard , and A. T. Fisk . 2010. “Diet and Resource Use Among Greenland Sharks (*Somniosus microcephalus*) and Teleosts Sampled in Icelandic Waters, Using d13 C, d15 N, and Mercury.” Canadian Journal of Fisheries and Aquatic Sciences 67: 1428–1438. 10.1139/F10-072.

[ece371564-bib-0040] Mecklenburg, C. W. , A. Lynghammar , E. Johannesen , et al. 2018. Marine Fishes of the Arctic Region. Conservation of Arctic Flora and Fauna.

[ece371564-bib-0041] Mortensen, J. , K. Lennert , J. Bendtsen , and S. Rysgaard . 2011. “Heat Sources for Glacial Melt in a Sub‐Arctic Fjord (Godthåbsfjord) in Contact With the Greenland Ice Sheet.” Journal of Geophysical Research: Oceans 116, no. C1: C01013. 10.1029/2010JC006528.

[ece371564-bib-0042] Mortensen, J. , S. Rysgaard , K. E. Arendt , et al. 2018. “Local Coastal Water Masses Control Heat Levels in a West Greenland Tidewater Outlet Glacier Fjord.” Journal of Geophysical Research: Oceans 123, no. 11: 8068–8083. 10.1029/2018JC014549.

[ece371564-bib-0043] Mortensen, J. , S. Rysgaard , M. H. S. Winding , et al. 2022. “Multidecadal Water Mass Dynamics on the West Greenland Shelf.” Journal of Geophysical Research: Oceans 127. 10.1029/2022JC018724.

[ece371564-bib-0044] Moura, T. , E. Jones , M. W. Clarke , et al. 2014. “Large‐Scale Distribution of Three Deep‐Water Squaloid Sharks: Integrating Data on Sex, Maturity and Environment.” Fisheries Research 157: 47–61. 10.1016/j.fishres.2014.03.019.

[ece371564-bib-0045] Mull, C. G. , and C. G. Lowe . 2010. “Seasonal Reproduction of Female Round Stingrays (*Urobatis halleri*): Steroid Hormone Profiles and Assessing Reproductive State.” General and Comparative Endocrinology 166, no. 2: 379–387. 10.1016/j.ygcen.2009.12.009. 20015450

[ece371564-bib-0046] Nielsen, J. 2018. “The Greenland Shark (Somniosus microcephalus). Diet, Tracking and Radiocarbon Age Estimates Reveal the World's Oldest Vertebrate.” PhD diss., University of Copenhagen.

[ece371564-bib-0048] Nielsen, J. , J. S. Christiansen , P. Grønkjær , et al. 2019a. “Greenland Shark (*Somniosus microcephalus*) Stomach Contents and Stable Isotope Values Reveal an Ontogenetic Dietary Shift.” Frontiers in Marine Science 6, no. 125. 10.3389/fmars.2019.00125.

[ece371564-bib-0047] Nielsen, J. , H. Carl , and P. R. Møller . 2019b. “Grønlandshaj/Havkal *Somniosus microcephalus* (Bloch & Schneider, 1801).” In Atlas Over danske saltvandsfisk, edited by H. Carl and P. R. Møller . Statens Naturhistoriske Museum. In Danish. https://fiskeatlas.ku.dk/artstekster/Gr_nlandshaj_Fiskeatlas.pdf.

[ece371564-bib-0049] Nielsen, J. , R. B. Hedeholm , J. Heinemeier , et al. 2016. “Eye Lens Radiocarbon Reveals Centuries of Longevity in the Greenland Shark (*Somniosus microcephalus*).” Science 353, no. 6300: 702–704. 10.1126/science.aaf3617. 27516602

[ece371564-bib-0050] Nielsen, J. , R. B. Hedeholm , A. Lynghammar , et al. 2020. “Assessing the Reproductive Biology of the Greenland Shark (*Somniosus microcephalus*).” PLoS One: 1–22. 10.1371/journal.pone.0238986. PMC7540863 33027263

[ece371564-bib-0051] Nielsen, J. , R. B. Hedeholm , M. Simon , and J. F. Steffensen . 2014. “Distribution and Feeding Ecology of the Greenland Shark ( *Somniosus microcephalus* ) in Greenland Waters.” Polar Biology 37, no. 1: 37–46. 10.1007/s00300-013-1408-3.

[ece371564-bib-0052] Nogueira, A. , H. Christensen , and J. Boje . 2023. “Survey for Greenland Halibut in ICES Division 14b, September‐October 2022 (Working Document NWWG (North Western Working Group) 24th April–28th April 2023).”

[ece371564-bib-0053] Nogueira, A. , and D. Estévez‐Barcia . 2023. “Results for Greenland Halibut Survey in NAFO Divisions 1C‐1D for the Period 1997–2017, 2019 and 2022 (NAFO Scientific Council Summary Document 23/012).”

[ece371564-bib-0054] Nygaard, R. 2020. “Trawl, Gillnet and Longline Survey Results From Surveys Conducted by the Greenland Institute of Natural Resources in NAFO Division 1A Inshore (NAFO Scientific Council Research Document 20/016).”

[ece371564-bib-0055] Nygaard, R. , and A. Nogueira . 2023. “Biomass and Abundance of Demersal Fish Stocks Off West Greenland Estimated From the Greenland Shrimp and Fish Survey 1990–2020 and 2022 (NAFO Scientific Council Research Document 23/020.).”

[ece371564-bib-0056] Orlov, A. M. , and S. Y. Orlova . 2024. “Eastward Journey: A Second Capture and First Genetically Confirmed Record of Greenland Shark *Somniosus microcephalus* in the Laptev Sea (Siberian Arctic).” Environmental Biology of Fishes 107: 47–57. 10.1007/s10641-024-01509-2.

[ece371564-bib-0057] Polloni, P. , R. Haedrich , G. Rowe , and C. H. Clifford . 1979. “The Size‐Depth Relationship in Deep Ocean Animals.” International Review of Hydrobiology 64: 39–46. 10.1002/iroh.19790640103.

[ece371564-bib-0058] Porteiro, F. , T. Sutton , I. Byrkjedal , et al. 2017. “Fishes of the Northern Mid‐Atlantic Ridge Collected During the MAR‐ECO Cruise in June–July 2004: An Annotated Checklist.” Arquipelago 126. http://nsuworks.nova.edu/occ_facreports/102.

[ece371564-bib-0059] Pratt, H. L. , T. C. Pratt , R. J. Knotek , J. C. Carrier , and N. M. Whitney . 2022. “Long‐Term Use of a Shark Breeding Ground: Three Decades of Mating Site Fidelity in the Nurse Shark, *Ginglymostoma cirratum* .” PLoS One 17, no. 10: e0275323. 10.1371/journal.pone.0275323. 36251734 PMC9576040

[ece371564-bib-0060] R Core Team . 2023. R: A Language and Environment for Statistical Computing. R Foundation for Statistical Computing. https://www.r‐project.org/.

[ece371564-bib-0061] Rideout, R. M. , B. Rogers , L. Wheeland , and M. Koen‐Alonso . 2022. “Temporal and Spatial Coverage of Canadian (Newfoundland and Labrador Region) Spring and Autumn Multi‐Species RV Bottom Trawl Surveys, With an Emphasis on Surveys Conducted in 2021 (NAFO Scientific Council Research Document; No. 22/007).”

[ece371564-bib-0062] Rusyaev, S. M. , and A. M. Orlov . 2013. “Bycatches of the Greenland Shark *Somniosus microcephalus* (Squaliformes, Chondrichthyes) in the Barents Sea and the Adjacent Waters Under Bottom Trawling Data.” Journal of Ichthyology 53, no. 1: 111–115. 10.1134/S0032945213010128.

[ece371564-bib-0063] Sætre, R. 2007. The Norwegian Coastal Current—Oceanography and Climate, edited by R. Sætre . Institute of Marine Research.

[ece371564-bib-0064] Sims, D. W. 2006. “Differences in Habitat Selection and Reproductive Strategies of Male and Female Sharks.” In Sexual Segregation in Vertebrates, edited by K. Ruckstuhl and P. Neuhaus , 127–147. Cambridge University Press. 10.1017/CBO9780511525629.009.

[ece371564-bib-0077] Skomal, G. B. , and G. W. Benz . 2004. “Ultrasonic Tracking of Greenland Sharks, *Somniosus microcephalus*, Under Arctic Ice.” Marine Biology 145, no. 3: 489–498. 10.1007/s00227-004-1332-8.

[ece371564-bib-0065] Straneo, F. , G. S. Hamilton , D. A. Sutherland , et al. 2010. “Rapid Circulation of Warm Subtropical Waters in a Major Glacial Fjord in East Greenland.” Nature Geoscience 3: 182–186. 10.1038/ngeo764.

[ece371564-bib-0066] Sulikowski, J. A. , C. R. Wheeler , A. J. Gallagher , B. K. Prohaska , J. A. Langan , and N. Hammerschlag . 2016. “Seasonal and Life‐Stage Variation in the Reproductive Ecology of a Marine Apex Predator, the Tiger Shark *Galeocerdo cuvier* , at a Protected Female‐Dominated Site.” Aquatic Biology 24: 175–184. 10.3354/ab00648.

[ece371564-bib-0067] Watanabe, Y. Y. , C. Lydersen , A. T. Fisk , and K. M. Kovacs . 2012. “Journal of Experimental Marine Biology and Ecology the Slowest Fish: Swim Speed and Tail‐Beat Frequency of Greenland Sharks.” Journal of Experimental Marine Biology and Ecology 426–427: 5–11. 10.1016/j.jembe.2012.04.021.

[ece371564-bib-0068] White, H. 1980. “A Heteroskedasticity‐Consistent Covariance Matrix Estimator and a Direct Test for Heteroskedasticity.” Econometrica 48: 817–838. 10.2307/1912934.

[ece371564-bib-0070] Yan, Y. , E. Cantoni , C. Field , M. Treble , and J. M. Flemming . 2022. “Spatiotemporal Modeling of Bycatch Data: Methods and a Practical Guide Through a Case Study in a Canadian Arctic Fishery.” Canadian Journal of Fisheries and Aquatic Sciences 79: 148–158. 10.1139/cjfas-2020-0267.

[ece371564-bib-0071] Yano, K. 1995. “Reproductive Biology of the Black Dogfish *Centroscyllium fabricii*, Collected From Waters Off Western Greenland.” Journal of the Marine Biological Association of the United Kingdom 75: 285–310.

[ece371564-bib-0072] Yano, K. , J. D. Stevens , and L. J. V. Compagno . 2007. “Distribution, Reproduction and Feeding of the Greenland Shark Somniosus (Somniosus) Microcephalus, With Notes on Two Other Sleeper Sharks, Somniosus (Somniosus) Pacificus and Somniosus (Somniosus) Antarcticus.” Journal of Fish Biology 70, no. 2: 374–390. 10.1111/j.1095-8649.2007.01308.x.

[ece371564-bib-0073] Yano, K. , and S. Tanaka . 1988. “Size at Maturity, Reproductive Cycle, Fecundity, and Depth Segregation of the Deep Sea Squaloid Sharks *Centroscymnus owstoni* and *C. coelolepis* in Suruga Bay, Japan.” Nippon Suisan Gakkaishi 54, no. 2: 167–174.

