# Supplementary material for: Spatial Distribution of Greenland Shark Somniosus microcephalus (Bloch & Schneider, 1801) Life Stages Across the Northern North Atlantic
Source: Ecol Evol. 2025 Jun 29;15(7):e71564. doi: 10.1002/ece3.71564 (PMC12206561; doi:10.1002/ece3.71564)
Supplement: Supplementary file 1 — Appendix S1. [file ECE3-15-e71564-s001.docx]

**Appendix 1: Gear and data source assumptions**

The data sources used in our study represent great variety in terms of sampling gear and protocols, which can raise concerns of potential bias. Greenland sharks (n=1,610) were sampled using bottom trawl (n=804), shark line (n=497), video (n=93), fishing rod (n=125, intended for deep sea fishes incl. Greenland shark), gill nets (n=78), and long lines (n=13) targeting Greenland halibut (**Table 1**). There was no significant effect of body size and gear type (ANOVA, F_15,1593_=1.07, P=0.37), yet it is relevant to consider whether certain gear types or data sources might miss particular life stages for example the smallest sizes (neonates and juveniles).


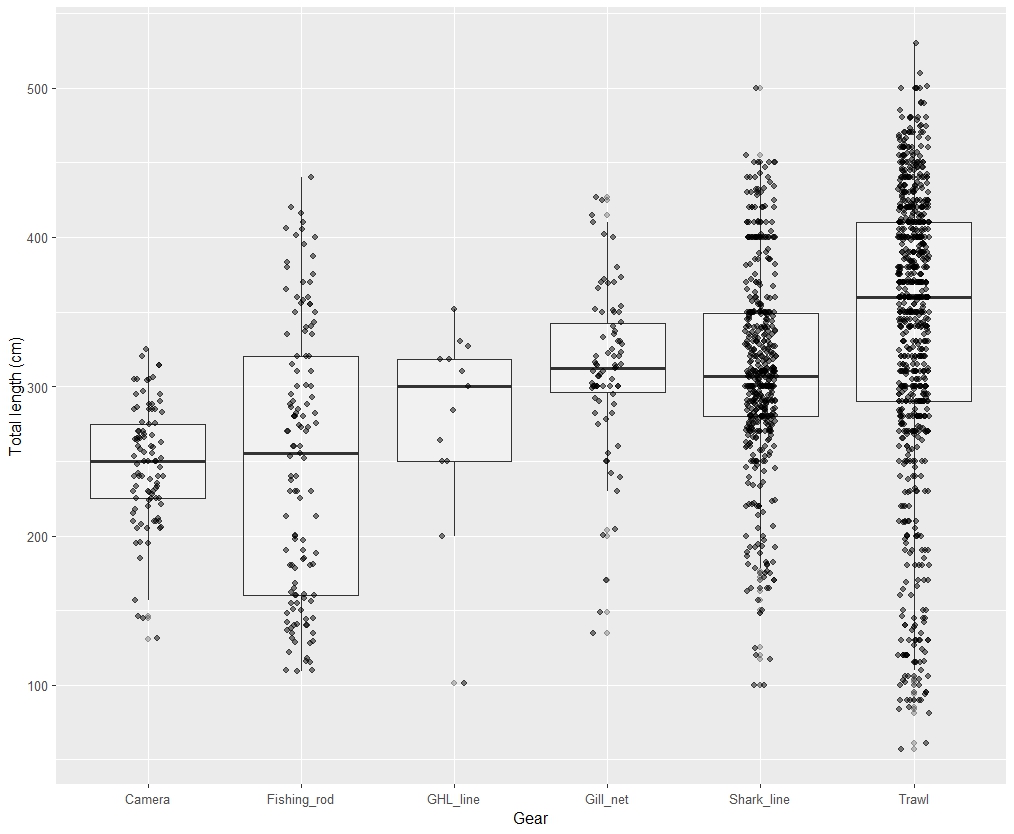


**Figure.** Box plots and associated data showing total length (cm) of the different sharks by gear. n=1,610. GHL_line=long line targeting Greenland halibut.

Greenland shark may vary in body size from neonates of total length ~0.4 m to mature females of at least TL 5.5 m. Scientific surveys utilizing bottom trawl, video, gill nets, and longlines intended for Greenland halibut have recorded very small body sizes of other teleosts and elasmobranchs, ranging from just a few centimeters up to approximately 30-40 cm (data not shown). Collectively, we expect these gear types to be capable of sampling the full range of Greenland shark body sizes and they represent 61 % of the evaluated dataset (N=988). The remaining 39 % of the dataset were caught by specially designed Greenland shark long lines (N=497) in scientific research programs or by fishing rod of recreational anglers (N=125). Neonates and juvenile Greenland sharks (TL<2 m) could potentially be underrepresented by these specific sampling gears, especially for shark lines that typically deploy large hooks and large pieces of bait (see example of shark line description in Nielsen et al. 2018). However, only two regions are composed by data from only shark lines (northeast Greenland, NEG) or fishing rod (Skagerrak, SKA). Although strong conclusions should not be made for these regions on the potential presence of neonates nor small juveniles here, trawl activity do occur in these areas yet the lack of reports in the scientific literature or in survey databases from Denmark, Sweden and Norway (data not show) strongly indicate that neonates are not present here We emphasize though that the analyzed dataset does not allow for strong conclusions in these two particular areas of SKA and EGN in terms of these smallest sizes of Greenland sharks. For all other regions, sampling gears without obvious size selectivity have been used, likely capturing the smallest sharks if they were commonly present in the investigated areas. Similarly, bottom trawls also sample the pelagic environment upon trawl deployment/retrieval. According to rough estimates from Greenland trawl surveys, deployment and retrieval take about the same time as effective fishing time on the bottom in standardized trawl surveys monitoring shrimp, Atlantic cod, or Greenland halibut. Therefore, a bottom trawl would also capture small Greenland sharks in the water column if they were present, similar to how trawls catch lanternfish and many other pelagic fishes (GINR unpublished data).

For the scientific literature, it is also assumed that sharks were randomly included in the respective studies with no bias in terms of size or sex. We found this to be a valid assumption for a range of feeding ecology studies (Leclerc et al. 2012, McMeans et al. 2010, Nielsen et 2019), tagging studies (Skomal & Benz 2004, Campana et al. 2015, Edwards et al. 2022, Fisk et al. 2012, Hussey et al. 2018, Nielsen 2018), abundance studies (Devine et al. 2018, Devine et al. unpublished, Hussey et al. 2015) and a reproduction study (Nielsen et al. 2020). Sharks from MacNeil et al., (2012), Nielsen et al., (2014), Nielsen et al. 2016, Ona & Nielsen, (2022), Strid et al., (2007, 2013), and Watanabe et al., (2012) are not included in this study as the specific individuals were included in the above mentioned studies. Also, records from museum collections could not be included for statistical analysis as such individuals tend to have been collected due to their (often) small size and therefore the assumption of random sampling cannot be fulfilled for such data source. Sharks from museum collections were however included in an additional effort to identify the location of rare neonates (TL<60 cm). Also, reports of rare neonates made by commercial pelagic fishermen to the HAFRO were included for biological evaluation although such data is neither sampled/recorded randomly. Lastly, Rusyaev & Orlov, (2013) and Yano et al., (2007) were not included (despite being sampled/recorded randomly), as the available data format did not allow individual information to be extracted.

In order to optimally assess the spatial distribution of life stages, it would be ideal to have data on the reproductive stage based on internal assessment for each shark. We encourage future studies and surveys to conduct internal assessments of maturation stage using the Greenland shark sex-specific maturity scale as proposed by Nielsen et al. 2020, if the shark cannot be released alive. Such data would allow for precise categorization of maturation ensuring that very large, but biologically subadult individuals (not sexually mature), are not mistakenly assigned as “adults”, as inevitably may occur applying a length-based approach, as done here. Nonetheless, for the purpose of the current study, length-based data serves as a sufficient proxy to estimate life stage. Our study aimed to provide novel insights based on demographic distributions in an attempt to more accurately “connect the dots” and better assess the puzzle of the Greenland shark life history than previously achieved.
