# Supplementary material for: Spatial Distribution of Greenland Shark Somniosus microcephalus (Bloch & Schneider, 1801) Life Stages Across the Northern North Atlantic
Source: Ecol Evol. 2025 Jun 29;15(7):e71564. doi: 10.1002/ece3.71564 (PMC12206561; doi:10.1002/ece3.71564)
Supplement: Supplementary file 2 — Appendix S2. [file ECE3-15-e71564-s004.docx]

**Appendix 2 – Data sources**

Campana, S. E., Fisk, A. T., & Klimley, A. P. (2015). Movements of Arctic and northwest Atlantic Greenland sharks (*Somniosus microcephalus*) monitored with archival satellite pop-up tags suggest long-range migrations. *Deep-Sea Research Part II*, *115*, 109–115. https://doi.org/10.1016/j.dsr2.2013.11.001

Devine, B. M., Wheeland, L. J., & Fisher, J. A. D. (2018). First estimates of Greenland shark (*Somniosus microcephalus*) local abundances in Arctic waters. *Scientific Reports*, *8*(974), 1–10. https://doi.org/10.1038/s41598-017-19115-x

Edwards, J. E., Edwards, J. E., Hedges, K. J., Kessel, S. T., & Hussey, N. E. (2022). Multi-year acoustic tracking reveals transient movements , recurring hotspots , and apparent seasonality in the coastal-offshore presence of Greenland sharks (*Somniosus microcephalus*). *Frontiers in Marine Science*, *September*, 1–17.

https://doi.org/10.3389/fmars.2022.902854

Fisk, A. T., Lydersen, C., & Kovacs, K. M. (2012). Archival pop-off tag tracking of Greenland sharks *Somniosus microcephalus* in the high Arctic waters of Svalbard, Norway. *Marine Ecoogy Progress Series*, *468*, 255–265.

https://doi.org/10.3354/meps09962

Hussey, N. E., Aurelie, C.-G., Walter, R. P., Hedges, K. J., Kessel, S. T., & Fisk, A. T. (2015). Juvenile Greenland sharks *Somniosus microcephalus* (Bloch & Schneider , 1801) in the Canadian Arctic. *Polar Biology*, *38*, 493–504.

https://doi.org/10.1007/s00300-014-1610-y

Hussey, N. E., Hedges, K. J., & Barkley, A. (2018). Mark report satellite tags (mrPATs) to detail large-scale horizontal movements of deep water species: first results for the Greenland shark (*Somniosus microcephalus*). *Deep-Sea Research Part I*, *134*, 32–40. https://doi.org/10.1016/j.dsr.2018.03.002

Leclerc, L. M. E., Lydersen, C., Haug, T., Bachmann, L., Fisk, A. T., & Kovacs, K. M. (2012). A missing piece in the Arctic food web puzzle? Stomach contents of Greenland sharks sampled in Svalbard, Norway. *Polar Biology*, *35*(8), 1197–1208.

https://doi.org/10.1007/s00300-012-1166-7

McMeans, B. C., Svavarsson, J., Dennard, S., & Fisk, A. T. (2010). Diet and resource use among Greenland sharks (*Somniosus microcephalus*) and teleosts sampled in Icelandic waters , using d 13 C , d 15 N , and mercury. *Canadian Journal of Fisheries and Aquatic Sciences*, *67*, 1428–1438. https://doi.org/10.1139/F10-072

Nielsen, J., Christiansen, J. S., Grønkjær, P., Bushnell, P., Steffensen, J. F., Kiilerich, H. O., Præbel, K., & Hedeholm, R. (2019). Greenland shark (*Somniosus microcephalus*) stomach contents and stable isotope values reveal an ontogenetic dietary shift. *Frontiers in Marine Science*, *6*(125). https://doi.org/10.3389/fmars.2019.00125

Nielsen, J., Hedeholm, R. B., Lynghammar, A., McClusky, L. M., Berland, B., Steffensen, J. F., & Christiansen, J. S. (2020). Assessing the reproductive biology of the Greenland shark (*Somniosus microcephalus*). *PLOS ONE*, 1–22.

https://doi.org/10.1371/journal.pone.0238986

Skomal, G. B., & Benz, G. W. (2004). Ultrasonic tracking of Greenland sharks, *Somniosus microcephalus*, under Arctic ice. *Marine Biology*, *145*(3), 489–498.

https://doi.org/10.1007/s00227-004-1332-8
