# Supplementary material for: Spatial Distribution of Greenland Shark Somniosus microcephalus (Bloch & Schneider, 1801) Life Stages Across the Northern North Atlantic
Source: Ecol Evol. 2025 Jun 29;15(7):e71564. doi: 10.1002/ece3.71564 (PMC12206561; doi:10.1002/ece3.71564)
Supplement: Supplementary file 3 — Figure S1. [file ECE3-15-e71564-s005.docx]

**Supplementary figures**


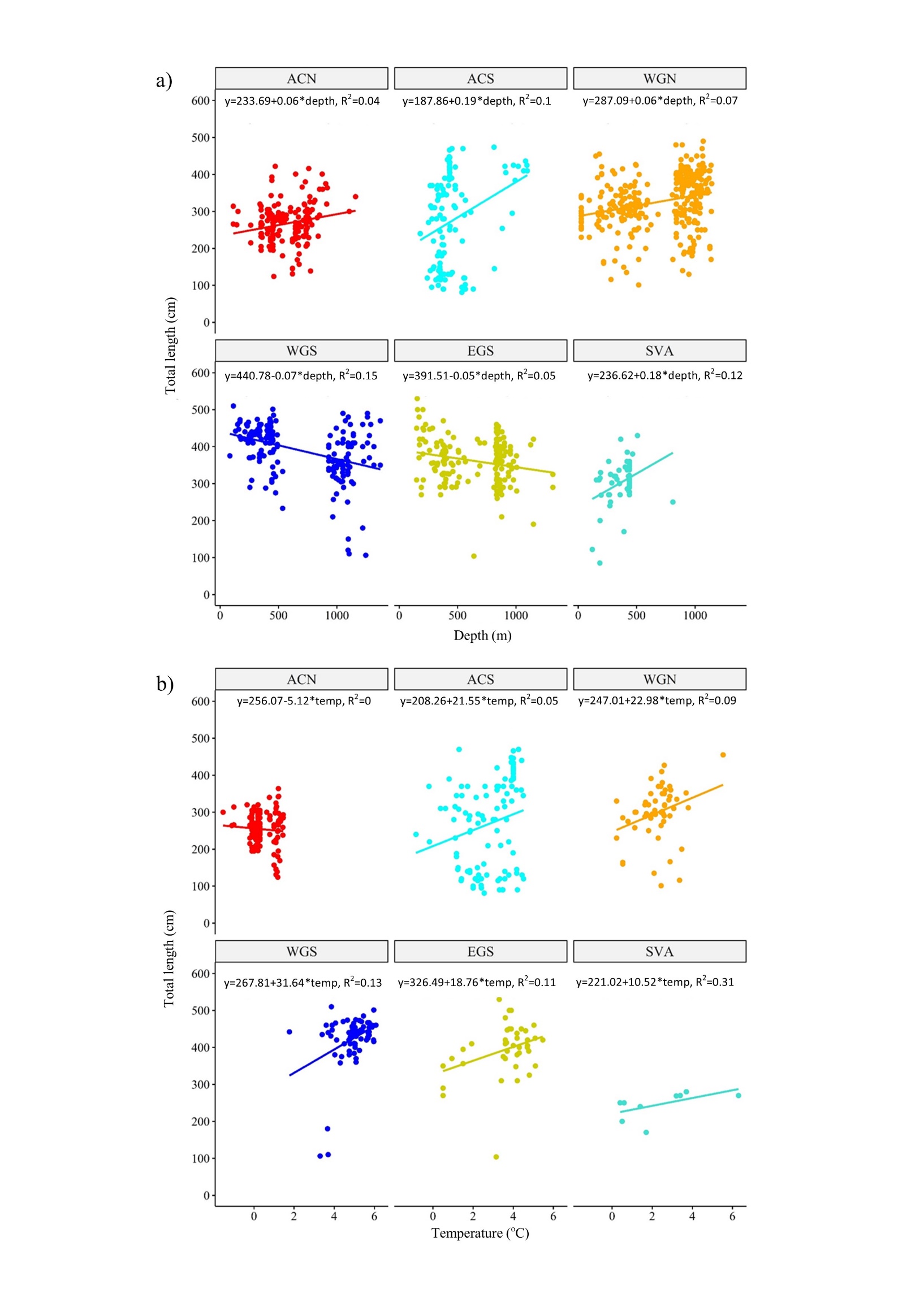


**Fig. S1:** Relationship between capture depth and total length for the six data-rich regions. The regression equation is provided for each plot. ACN=Arctic Canada north; ACS=Arctic Canada south; WGN=West Greenland north; WGS=West Greenland south; EGS=East Greenland south.


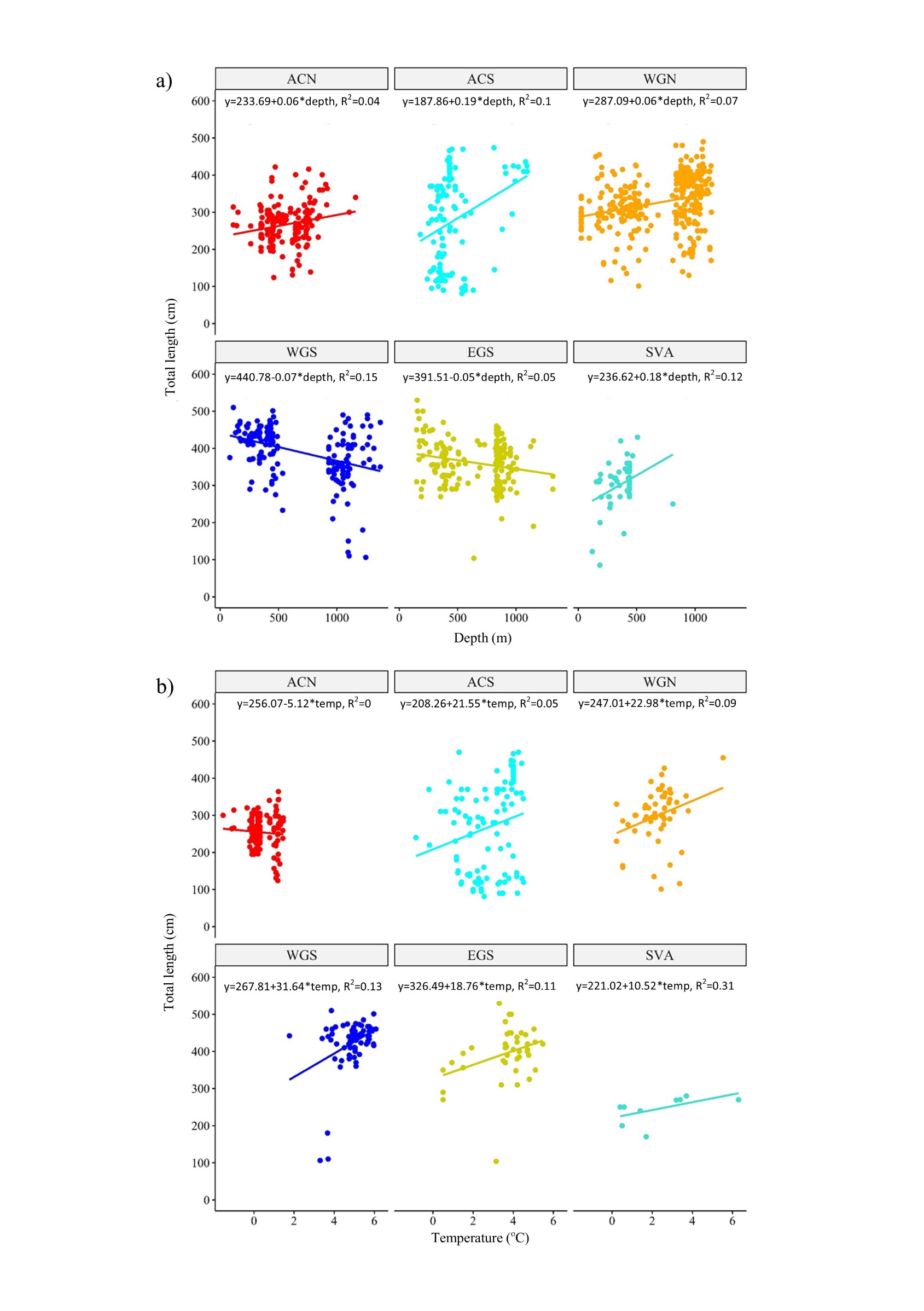


**Fig. S2:** Relationship between temperature (^o^C) and total length for nine regions. The regression equation is provided for each plot. ACN=Arctic Canada north; ACS=Arctic Canada south; WGN=West Greenland north; WGS=West Greenland south; EGS=East Greenland south; ICE=Iceland; FAR=Faroe Islands; NOR=Norway mainland.
