# Supplementary material for: Spatial Distribution of Greenland Shark Somniosus microcephalus (Bloch & Schneider, 1801) Life Stages Across the Northern North Atlantic
Source: Ecol Evol. 2025 Jun 29;15(7):e71564. doi: 10.1002/ece3.71564 (PMC12206561; doi:10.1002/ece3.71564)
Supplement: Supplementary file 5 — Table S2. [file ECE3-15-e71564-s003.docx]

**Table S2:** Contingency table showing the sample size by region and the expected values for the chi-square test.

|  |  |  | | |  |  | | |
| --- | --- | --- | --- | --- | --- | --- | --- | --- |
|  |  | **Sample size by region** | | |  | **Expected values** | | |
|  | Total | Large | Medium | Small |  | Large | Medium | Small |
| **ACN** | 269 | 4 | 222 | 43 |  | 49 | 191 | 29 |
| **ACS** | 132 | 25 | 66 | 41 |  | 24 | 94 | 14 |
| **EGN** | 12 | 0 | 12 | 0 |  | 2 | 9 | 1 |
| **EGS** | 164 | 40 | 122 | 2 |  | 30 | 116 | 18 |
| **FAR** | 17 | 7 | 8 | 2 |  | 3 | 12 | 2 |
| **ICE** | 55 | 29 | 19 | 7 |  | 10 | 39 | 6 |
| **NOR** | 76 | 8 | 61 | 7 |  | 14 | 54 | 8 |
| **SKA** | 66 | 1 | 18 | 47 |  | 12 | 47 | 7 |
| **SVA** | 119 | 3 | 113 | 3 |  | 22 | 84 | 13 |
| **WGN** | 406 | 51 | 337 | 18 |  | 74 | 288 | 44 |
| **WGS** | 294 | 125 | 163 | 6 |  | 54 | 208 | 32 |
|  |  |  |  |  |  |  |  |  |
| Total | 1610 | 293 | 1141 | 176 |  | 293 | 1141 | 176 |
|  |  |  |  |  |  |  |  |  |
